# Partial regularity for minima of higher-order quasiconvex integrands with natural Orlicz growth

**DOI:** 10.1007/s10231-025-01547-2

**Published:** 2025-01-24

**Authors:** Christopher Irving

**Affiliations:** Faculty of Mathematics, Technical University Dortmund, Vogelpothsweg 87, 44227 Dortmund, Germany

**Keywords:** Higher-order quasiconvexity, Epsilon-regularity, General growth, 35J48, 35J50

## Abstract

A partial regularity theorem is presented for minimisers of $k^{\textrm{th}}$-order functionals subject to a quasiconvexity and general growth condition. We will assume a natural growth condition governed by an *N*-function satisfying the $\Delta _2$ and $\nabla _2$ conditions, assuming no quantitative estimates on the second derivative of the integrand; this is new even in the $k=1$ case. These results will also be extended to the case of strong local minimisers.

## Introduction

In this paper we will investigate the regularity of minimisers of functionals of the form

1.1 $$\begin{aligned} {\mathcal {F}}(w) = \int _{\Omega } F(\nabla ^k w) \,\textrm{d}x, \end{aligned}$$

where $\Omega \subset \mathbb R^n$ is a bounded domain and $w :\Omega \rightarrow \mathbb R^N$ is a vector-valued mapping. We will assume *F* satisfies a suitable *higher-order quasiconvexity condition* in the sense of Morrey [55] for $k=1$ and Meyers [53] for $k\ge 2,$ and a general growth condition governed by an *N*-function $\varphi$ which we specify below. If *F* is sufficiently regular, it is known that minimisers *u* of (1.1) are *F*-*extremal,* that is they satisfy the associated Euler-Lagrange system

1.2 $$\begin{aligned} (-1)^k \nabla ^k: F'(\nabla ^k u):= (-1)^k\sum _{|\alpha |=k} \textrm{D}^{\alpha } \left( F'(\nabla ^ku): \mathrm e^{\alpha }\right) = 0 \end{aligned}$$

in $\Omega ,$ using the notation detailed in Sect. 2.1.

A striking feature of vector-valued problems is that minimisers need not be regular in general (see for instance [20, 52, 54, 58, 66]), however we can still hope for *partial regularity results*, which assert that, away from a small singular set, minimisers *u* are as regular as the data allows. The first partial regularity result in the quasiconvex setting was established by Evans [30] which has been extended significantly since; see [2, 3, 9, 13, 18, 19, 22, 29, 33, 34, 39, 48, 49] for a non-exhaustive list.

In the higher order case, the regularity theory for strongly *k*-quasiconvex integrands has been studied for instance by Guidorzi [42], Kronz [49], and Schemm [61]. Note the results of [10, 17] show that higher-order quasiconvexity reduces to ordinary quasiconvexity under quantitative Lipschitz bounds on $F',$ and while these results cannot be directly applied, we expect the higher order case to be essentially the same as the $k=1$ setting.

We will mention that in the quasiconvex setting, some kind of minimising condition is essential to obtain regularity in general. This is illustrated in the seminal work of Müller & Šverák [57] where Lipschitz but nowhere ${{\,\textrm{C}\,}}^1$ solutions to the associated Euler-Lagrange system are constructed, and this has been extended by Kristensen & Taheri [48] and Székelyhidi [67] to weak local minimisers and strongly polyconvex integrands respectively. As illustrated in [48] one can infer partial regularity for strong local minimisers however, which we discuss in Sect. 4.

### Hypotheses and main results

We will consider higher-order integrands satisfying a natural growth condition governed by an *N*-function, and a strict quasiconvexity condition which matches said growth bound. We refer the reader to Sects. 2.1, 2.2 for the precise definitions and conventions we use.

#### Hypotheses 1.1

Let $F :\mathbb M_k \rightarrow \mathbb R$ with $n \ge 2,$ $N,k\ge 1$ satisfy the following.

(H0) *F* is $${{\,\textrm{C}\,}}^2$$ regular.
(H1) There exists $K \ge 0$ and an *N*-function $\varphi$ satisfying the $\Delta _2$ and $\nabla _2$ conditions (as defined in Sect. 2.2) such that
  $$\begin{aligned} |F(z)|\le K \left( 1 + \varphi (|z|) \right) \end{aligned}$$
  for all $z \in \mathbb M_k.$ By rescaling $\varphi , K$ if necessary, we will assume $\varphi (1)=1.$
(H2) *F* is *strictly* ${{\,\textrm{W}\,}}^{k,\varphi }$-*quasiconvex* in the sense that there is $\nu >0$ such that
  $$\begin{aligned} \int _{\mathbb R^n} F(z_0 + \nabla ^k \xi ) - F(z_0) \,d x \ge \nu \int _{\mathbb R^n} \varphi _{1+|z_0|}(|\nabla ^k \xi |) \,\textrm{d}x \end{aligned}$$
  for all $z_0 \in \mathbb M_k$ and $\xi \in {{\,\textrm{C}\,}}^{\infty }_c(\mathbb R^n),$ where $\varphi _a$ is defined in (2.8).

For integrands of this type, we say $u \in {{\,\textrm{W}\,}}^{k,\varphi }(\Omega ,\mathbb R^N)$ is a *minimiser* of (1.1) if for any $\xi \in {{\,\textrm{W}\,}}^{k,\varphi }_0(\Omega ,\mathbb R^N)$ we have

1.3 $$\begin{aligned} {\mathcal {F}}(u) \le {\mathcal {F}}(u+\xi ). \end{aligned}$$

Note that it suffices to verify this for $\xi \in {{\,\textrm{C}\,}}^{\infty }_c(\Omega ,\mathbb R^N),$ from which the above follows by density (using both (H1) and (H2)).

We will point out that, while the partial regularity for quasiconvex integrands satisfying a general growth condition have been considered by Diening, Lengeler, Stroffolini, & Verde [22] for the $k=1$ case, the authors assume a *controlled growth condition* where quantitative estimates are assumed on the second derivatives $F''.$ We will relax this condition and establish the following.

#### Theorem 1.2

($\varepsilon$-regularity theorem) Let *F* satisfy Hypotheses 1.1 and let $M>0,$ $\alpha \in (0,1)$ be given. Then there exists $\varepsilon >0$ such that if $u \in {{\,\textrm{W}\,}}^{k,\varphi }(\Omega ,\mathbb R^N)$ minimises (1.1) where $\Omega \subset \mathbb R^n$ is a bounded domain, and $\textrm{B}_R(x_0) \subset \Omega$ is such that1.4then we have *u* is of class ${{\,\textrm{C}\,}}^{k,\alpha }$ in $\textrm{B}_{R/2}(x_0).$

Our proof largely hinges on a suitable Caccioppoli inequality and a harmonic approximation argument, which can be traced back to the works of Giusti & Miranda [37] and Morrey [56] in the general variational context, which were inspired by prior works in the geometric setting. The Caccioppoli inequality will be based on of the version in [30], which was adapted to the general growth setting in [22]. For the harmonic approximation argument we will adapt a recent approach of Gmeineder & Kristensen [39], which uses a duality argument to directly estimate the excess in the approximation. This approach has also been surveyed in the *p*-growth setting by Bärlin, Gmeineder, Kristensen and the author in [7].

To apply this harmonic approximation argument from [39] in the general growth setting, we will need to additionally use a version of the Lipschitz truncation lemma of Acerbi & Fusco [1, 2]. This approach parallels the ${\mathcal {A}}$-harmonic approximation argument which has appeared in for instance [24, 26–28, 49], which traces back to arguments from geometric problems and can be found in texts of Simon [63, 64]. In [22] a direct proof of a suitable ${\mathcal {A}}$-harmonic approximation is proven by applying a Lipschitz truncation to a suitable dual problem, which closely mirrors the strategy we adopt.

Once the above $\varepsilon$-regularity theorem is established, the following partial regularity theorem follows by standard means.

#### Theorem 1.3

(Partial regularity of minimisers) Let *F* satisfy Hypotheses 1.1, and $u \in {{\,\textrm{W}\,}}^{k,\varphi }(\Omega ,\mathbb R^N)$ be a minimiser of (1.1), where $\Omega \subset \mathbb R^N$ is a bounded domain. Then there exists an open subset $\Omega _0\subset \Omega$ of full measure such that *u* is ${{\,\textrm{C}\,}}^{k,\alpha }$ in $\Omega _0$ for each $\alpha \in (0,1).$ Moreover we have $\Omega _0 = \Omega {\setminus } \left( \Sigma _1 \cup \Sigma _2\right) ,$ where1.51.6

In light of the partial regularity results in the linear growth setting [39], a natural question is whether the $\nabla _2$-condition is really necessary. In fact the results in [41] (see also [40]) implies that partial regularity holds if we have merely the growth condition $\varphi (t) \le C t^q$ with $q < \frac{n}{n-1},$ however the general case remains open, which we wish to address in future work.

## Preliminaries

### Notation

We will briefly fix some notation which will be used throughout the text. We denote by $\mathbb M_k = {{\,\textrm{Sym}\,}}_k(\mathbb R^n,\mathbb R^N)$ the space of symmetric *k*-linear maps $(\mathbb R^n)^k \rightarrow \mathbb R^N,$ which is equipped with the inner product $z:w = \sum _{|\alpha |=k} z(\mathrm e^{\alpha })\cdot w(\mathrm e^{\alpha })$ for $z,w \in \mathbb M_k,$ taking tensor powers of the standard orthonormal basis $\{\mathrm e_i\}_{i=1}^n$ of $\mathbb R^n.$ The associated norm is denoted $|z|= \sqrt{z: z}.$ Note that we identify $\mathbb M_0 = {\mathbb {R}}^N$ as vectors in ${\mathbb {R}}^N$ and $\mathbb M_1 = \mathbb R^{Nn}$ as the space of $N \times n$ matrices. If $u :\Omega \rightarrow \mathbb R^N$ is *k*-times continuously differentiable where $\Omega \subset \mathbb R^n$ is an open set, we denote the partial derivatives of *u* by $\textrm{D}^{\alpha }u$ using multi-index notation, and its *k*^th^ order gradient by $\nabla ^k u :\Omega \rightarrow {\mathbb {M}}_k$, given by $\nabla ^ku(\mathrm e^{\alpha }) = \textrm{D}^{\alpha } u$. The same notation will be used for weak derivatives.

We will equip $\mathbb R^n$ with the Lebesgue measure ${\mathcal {L}}^n,$ and if $A \subset \mathbb R^n$ is non-empty and open such that $0<{\mathcal {L}}^n(A)<\infty$, for any $f \in {{\,\textrm{L}\,}}^1(A,\mathbb V)$ with $(\mathbb V,|\,\cdot \,|)$ a finite-dimensional real vector space, we define2.1We also denote by a $\textrm{B}_R(x_0)$ the open ball in $\mathbb R^n$ centred at $x_0$ with radius *R*.

For a differentiable map $F :\mathbb M_k \rightarrow \mathbb R$ we define its derivative $F' :\mathbb M_k \rightarrow \mathbb M_k$ as

2.2 $$\begin{aligned} F'(z)w = \left. \frac{\textrm{d}}{\textrm{d}t}\right| _{t=0} F(z+tw), \end{aligned}$$

and if *F* is ${{\,\textrm{C}\,}}^2,$ its second derivative $F''(z)$ will be a linear map $\mathbb M_k \rightarrow \mathbb M_k$ satisfying

2.3 $$\begin{aligned} F''(z)v: w = \left. \frac{\textrm{d}}{\textrm{d}t}\right| _{t=0} F'(z+tv) w. \end{aligned}$$

This can be viewed as a symmetric bilinear form on $\mathbb M_k.$

Additionally $C, C_1, C_2,\dots$ will denote constants which may change from line to line, and if not specified will depend only on the parameters the resulting estimate depends on. We may also write $C_{\alpha ,\beta ,\dots }$ to emphasise the dependence on certain parameters. We also write $A \sim B$ if there exists constants $C_1,C_2>0$ such that $C_1A \le B \le C_2A.$

### *N*-functions

We will define the scales of growth we are interested in, and record some basic properties. These results can be found for instance in [4, 47, 59].

We say $\varphi :[0,\infty ) \rightarrow [0,\infty )$ is an *N*-*function* if $\varphi$ is increasing, continuous and convex such that $\varphi (t)=0$ if and only if $t=0$ and we have the limits

2.4 $$\begin{aligned} \lim _{t \rightarrow 0} \frac{\varphi (t)}{t} = 0, \quad \lim _{t \rightarrow \infty } \frac{\varphi (t)}{t} = \infty \end{aligned}$$

hold true. Given such a $\varphi$, define its *conjugate function* by

2.5 $$\begin{aligned} \varphi ^*(s) = \int _0^s (\varphi ')^{-1}(\sigma )\,\textrm{d}\sigma = \int _0^s\inf \{\tau> 0 :\varphi '(\tau ) > \sigma \}\,\textrm{d}\sigma . \end{aligned}$$

We say an *N*-function $\varphi$ satisfies the $\Delta _2$-*condition* if there is $C \ge 1$ for which $\varphi (2t) \le C\varphi (t)$ holds for all $t\ge 0,$ and the least such *C* will be denoted $\Delta _2(\varphi ).$ We also say $\varphi$ satisfies the $\nabla _2$-*condition* if its conjugate $\varphi ^*$ satisfies the $\Delta _2$-condition, and write $\nabla _2(\varphi ) = \Delta _2(\varphi ^*).$ We will use the notation $\varphi \in \Delta _2,$ $\varphi \in \nabla _2$ to denote *N*-functions satisfying the $\Delta _2$ and $\nabla _2$ conditions respectively, and write $\varphi \in \Delta _2\cap \nabla _2$ if both are satisfied.

Note that if $\varphi \in \Delta _2,$ then there is $p>1$ such that $\varphi (st) \le s^{p} \varphi (t)$ for all $t> 0, s>1.$ The minimal such *p* will be denoted by $p_{\varphi }.$

#### Lemma 2.1

The following inequalities hold for an *N*-function $\varphi .$

- (Young’s inequality) For $t,s \ge 0$ we have
  $$\begin{aligned} ts \le \varphi (t) + \varphi ^*(s), \end{aligned}$$
  with equality if and only if $s= \varphi '(t)$ or $t=(\varphi ^*)'(s).$
- For any $t \ge 0$ we have
  $$\begin{aligned} t \le \varphi (t)^{-1}(\varphi ^*)^{-1}(t) \le 2t. \end{aligned}$$
- If $\varphi \in \Delta _2 \cap \nabla _2$ we have the equivalences
  $$\begin{aligned} \varphi (t) \sim t \varphi '(t) \sim \varphi ^*(\varphi '(t)) \sim \varphi ^*(\varphi (t)/t). \end{aligned}$$

We refer the reader to [59] for a proof; note that (a) and (b) are shown in Theorem I.3 and Proposition II.1(ii) respectively, and (c) follows by convexity of $\varphi$ and (b).

In particular (a) implies the equivalent definition

2.6 $$\begin{aligned} \varphi ^*(s) = \sup _{t>0} \left( ts - \varphi (t)\right) . \end{aligned}$$

Also for any $\delta >0$, applying (a) with $s/\delta$ in place of *s*, we have

2.7 $$\begin{aligned} ts \le \delta \varphi (t) + \delta \varphi ^*(s/\delta ). \end{aligned}$$

If $\varphi$ is an *N*-function and $a > 0,$ following [6] we also introduce the *shifted* *N*-*function*

2.8 $$\begin{aligned} \varphi _a(t) = \int _0^t \frac{\tau \varphi '(\max \{a,\tau \})}{\max \{a,\tau \}} \,\textrm{d}\tau . \end{aligned}$$

Note that $\varphi _a(t) \sim t^2$ if $t \le a$ and $\varphi _a(t) \sim \varphi (t)$ if $t\ge a$ (where the constant depends on *a*) and using (2.5) we have $(\varphi _a)^* = (\varphi ^*)_{\varphi '(a)}.$ Further if $\varphi \in \Delta _2\cap \nabla _2,$ then the same holds for each $\varphi _a$ with $1 \le \Delta _2(\varphi _a) \le \Delta _2(\varphi ),$ $1 \le \nabla _2(\varphi _a) \le \nabla _2(\varphi )$ for all $a>0.$ Also if $\varphi \in \Delta _2,$ then for each $M>0$ we have $\varphi _{1+a} \sim \varphi _{1+M}$ for all $0 \le a \le M$ (where the constant depends on $M, \Delta _2(\varphi )$).

### Orlicz-Sobolev spaces

We will also define the natural function spaces associated to the functional (1.1), and establish some basic properties that we will use later.

#### Definition 2.2

For an open set $\Omega \subset \mathbb R^n,$ a finite-dimensional normed space $(\mathbb V,|\,\cdot \,|)$ and $\varphi \in \Delta _2,$ we define the *Orlicz space* ${{\,\textrm{L}\,}}^{\varphi }(\Omega ,\mathbb V)$ as the space of $f \in {{\,\textrm{L}\,}}^1_{{{\,\textrm{loc}\,}}}(\Omega ,\mathbb V)$ for which

2.9 $$\begin{aligned} \rho _{\Omega }^{\varphi }(|f|):= \int _{\Omega } \varphi (|f |) \,\textrm{d}x < \infty , \end{aligned}$$

which we equip with the *Luxemburg norm*

2.10 $$\begin{aligned} \left\Vert f\right\Vert _{{{\,\textrm{L}\,}}^{\varphi }(\Omega ,\mathbb V)} = \inf \left\{ \lambda >0 :\rho _{\Omega }^{\varphi }\left( \frac{|f|}{\lambda } \right) \le 1 \right\} . \end{aligned}$$

Note that the $\Delta _2$-condition ensures that ${{\,\textrm{L}\,}}^{\varphi }(\Omega ,\mathbb V)$ as defined is a linear space, and that $f \in {{\,\textrm{L}\,}}^1_{{{\,\textrm{loc}\,}}}(\Omega ,\mathbb V)$ lies in ${{\,\textrm{L}\,}}^{\varphi }(\Omega ,\mathbb V)$ if and only if $\left\Vert f\right\Vert _{{{\,\textrm{L}\,}}^{\varphi }(\Omega ,\mathbb V)} < \infty .$ Also if $\Omega$ is bounded, we have ${{\,\textrm{L}\,}}^{\varphi }(\Omega ,\mathbb V) = {{\,\textrm{L}\,}}^{\varphi _{a}}(\Omega ,\mathbb V)$ for all $a>0,$ with $\varphi _a$ defined as in (2.8).

Given this, for $N, k, \ell \ge 1$ we can define the *Orlicz-Sobolev spaces* ${{\,\textrm{W}\,}}^{k,\varphi }(\Omega ,\mathbb M_{\ell })$ as the space of *k*-times weakly differentiable mappings $u \in {{\,\textrm{L}\,}}^{\varphi }(\Omega ,\mathbb M_{\ell })$ such that $\nabla ^j u \in {{\,\textrm{L}\,}}^{\varphi }(\Omega ,\mathbb M_{\ell +j})$ for all $1 \le j \le k.$ We equip this space with the norm

2.11 $$\begin{aligned} \left\Vert u\right\Vert _{{{\,\textrm{W}\,}}^{k,\varphi }(\Omega ,\mathbb M_{\ell })} = \sum _{j=0}^{k} \left\Vert \nabla ^ju\right\Vert _{{{\,\textrm{L}\,}}^{\varphi }(\Omega ,\mathbb M_{\ell +j})}. \end{aligned}$$

We also define ${{\,\textrm{W}\,}}^{k,\varphi }_0(\Omega ,\mathbb M_{\ell })$ to be the closure of ${{\,\textrm{C}\,}}^{\infty }_c(\Omega ,\mathbb M_{\ell })$ with respect to $\left\Vert \cdot \right\Vert _{{{\,\textrm{W}\,}}^{k,\varphi }(\Omega ,\mathbb M_{\ell })},$ and ${{\,\textrm{W}\,}}^{k,\varphi }_{{{\,\textrm{loc}\,}}}(\Omega ,\mathbb M_{\ell })$ to be the space of $u \in {{\,\textrm{W}\,}}^{k,1}_{{{\,\textrm{loc}\,}}}(\Omega ,\mathbb M_{\ell })$ such that the restriction $u|_{\Omega '}$ lies in ${{\,\textrm{W}\,}}^{k,\varphi }(\Omega ',\mathbb M_{\ell })$ for each compactly contained domain $\Omega ' \Subset \Omega .$

These Orlicz-Sobolev spaces enjoy many of the familiar properties satisfied by the standard Sobolev spaces; see for instance [4, Section 8]. We will record some specific results we need here. This first result we need is the following Poincaré-Sobolev inequality, which is far from sharp, but will suffice for our purposes (for a sharp statement see [16]).

#### Lemma 2.3

Let $\varphi \in \Delta _2$ and $\ell \ge 1$, then if $u \in {{\,\textrm{W}\,}}^{1,\varphi }(\Omega ,\mathbb M_{\ell })$ we have $u \in {{\,\textrm{L}\,}}^{\varphi ^p}_{{{\,\textrm{loc}\,}}}(\Omega ,\mathbb M_{\ell })$ for each $1 \le p \le \frac{n}{n-1}.$ Moreover for any $\textrm{B}_R(x_0) \subset \Omega$ we have2.12where $C=C(n,\Delta _2(\varphi ))>0.$ If $u \in {{\,\textrm{W}\,}}^{1,\varphi }_0(\textrm{B}_R(x_0),\mathbb M_{\ell }),$ the same holds without subtracting the average.

#### Proof

We will establish the result for $R=1,$ from which the general case follows by rescaling. The result for $p=1$ follows by a standard application of the Riesz potential, as is shown in [8] (see also [21]). For general $1 \le p \le \frac{n}{n-1}$ assume that $(u)_{\textrm{B}_1(x_0)}=0$ by translation, and noting that $|\nabla (\varphi (|u |))|= \varphi '(|u|)|\nabla u|$ we can apply Young’s inequality to bound2.13From here we conclude that $\varphi (|u|) \in {{\,\textrm{W}\,}}^{1,1}(\textrm{B}_1(x_0))$ using the $\Delta _2$-condition and the $p=1$ case, and so by the Gagliardo-Nirenberg inequality we have $\varphi (|u|) \in {{\,\textrm{L}\,}}^p(\textrm{B}_1(x_0))$ for each $1 \le p \le \frac{n}{n-1}$ with the associated estimate2.14as required. The ${{\,\textrm{W}\,}}^{1,\varphi }_0(\Omega ;{\mathbb {M}}_{\ell })$ case can be found in [15, Proposition 4.1]. $\square$

#### Remark 2.4

Applying this iteratively with $p=1$, we deduce for all $0 \le j \le k-1$ that2.15where $a_{x_0,R}$ is the unique polynomial of degree at most $k-1$ satisfying2.16for all $|\alpha |\le k.$ Similarly as in the $k=1$ case, we can omit the $a_{j,u}$ term if $u \in {{\,\textrm{W}\,}}^{k,\varphi }_0(\textrm{B}_R(x_0),\mathbb M_{\ell })$.

We will also need some results for affine functions in Sect. 4, where we consider strong local minimisers. These can be deduced by combining the results in [43, Lemmas 2.2, 2.3] and [49, Lemma 2].

#### Lemma 2.5

Let $\textrm{B}_R(x_0)\subset \mathbb R^n$ be a ball, $N,\ell \ge 1,$ and $\varphi \in \Delta _2.$ Then if $u \in {{\,\textrm{W}\,}}^{1,\varphi }(\textrm{B}_R(x_0),\mathbb M_{\ell })$, we have the affine function $A_{x_0,R} :\mathbb R^n \rightarrow \mathbb M_{\ell }$ defined to satisfy2.17satisfies2.18for any other $A :\mathbb R^n \rightarrow \mathbb M_{\ell }$ affine, and we also have the estimates2.192.20for all $\sigma \in (0,1).$

Finally we record a interpolation estimate in the Orlicz scales, which is a straightforward adaption of the ${{\,\textrm{W}\,}}^{k,p}$ case (compare with the results in [4, Section 5]).

#### Lemma 2.6

Let $\varphi \in \Delta _2$ and $k \ge 0.$ Then there exists $C=C(n,k,\Delta _2(\varphi ))>0$ such that for all $x_0 \in \mathbb R^n,$ $R>0$ and $u \in {{\,\textrm{W}\,}}^{k,\varphi }(\textrm{B}_R(x_0),\mathbb M_{\ell }),$ we have the estimate

2.21 $$\begin{aligned} \int _{\textrm{B}_R(x_0)} \varphi (|\nabla ^ju|) \,\textrm{d}x \le C\int _{\textrm{B}_R(x_0)} \varphi (\delta ^{-j}|u|) + \varphi (\delta ^{k-j} |\nabla ^ku|) \,\textrm{d}x \end{aligned}$$

holds for all $\delta >0$.

#### Sketch of proof

We start by observing the one-dimensional estimate

2.22 $$\begin{aligned} \varphi (|f'(0)|) \le \frac{C}{\delta } \int _0^{\delta } \varphi (\delta ^{-1}|f(t)|) + \varphi (\delta |f''(t)|) \,\textrm{d}t, \end{aligned}$$

valid for $C^2$ functions $f :[0,\delta _0) \rightarrow {\mathbb {R}}$ and $\delta \le \delta _0$, following [4, Lemma 5.4] using Jensen’s inequality with $\varphi .$ We moreover observe that this inequality remains valid for all $\delta >0$, where we understand the integral on the right-hand side to vanish on $[\delta _0,\infty )$. Then assuming *u* is sufficiently regular, for $x \in \textrm{B}_R(x_0)$ and $\omega \in S^{n-1}$ we can apply this to $f(t) = u(x+t\omega )$. Integrating the obtained bound over $x, \omega$ gives

2.23 $$\begin{aligned} \begin{aligned} \int _{\textrm{B}_R(x_0)} \varphi (|\nabla u|) \,\textrm{d}x&\le C \int _{\textrm{B}_{R}(x_0)} \varphi \left( \delta ^{-1}|u(x)|\right) + \varphi \left( \delta |\nabla ^2u(x)|\right) \,\textrm{d}x, \end{aligned} \end{aligned}$$

nothing there is no contribution if $x + t\omega \notin \textrm{B}_R(x_0)$, thereby establishing the $j=1,$ $k=2$ case. For the general case we proceed by induction; suppose the result holds for some $k \ge 2$ and $j=k-1,$ then for $\delta >0$ and $\mu \in (0,1)$ we can estimate

2.24 $$\begin{aligned} \begin{aligned} \int _{\textrm{B}_R(x_0)} \varphi (|\nabla ^k u|) \,\textrm{d}x&\quad \le C \int _{\textrm{B}_R(x_0)} \varphi (\delta ^{-1}|\nabla ^{k-1} u|) + \varphi (\delta |\nabla ^{k+1}u|) \,\textrm{d}x\\&\quad \le C \int _{\textrm{B}_R(x_0)} \varphi ((\mu \delta )^{-1}\delta ^{-1}|u|) + \varphi (\mu |\nabla ^k u|) + \varphi (\delta |\nabla ^{k+1}u|) \,\textrm{d}x. \end{aligned}\nonumber \\ \end{aligned}$$

By convexity of $\varphi$ we can choose $\mu >0$ sufficiently small so that $C\varphi (\mu |\nabla ^k u|) \le \frac{1}{2} \varphi (|\nabla ^ku|)$, from which the estimate follows. A similar downward induction argument extends the result to all $1 \le j \le k-1.$ $\square$

### Linear elliptic estimates

Our linearisation strategy will involve comparing our minimiser with solutions to a linearised system, for which we will need some solvability results. We will consider a bilinear form $\mathbb A$ on $\mathbb M_k$ satisfying the *uniform Legendre-Hadamard ellipticity condition*

2.25 $$\begin{aligned} \lambda |\xi |^2 |\eta |^{2k} \le \mathbb A[\xi \otimes \eta ^k,\xi \otimes \eta ^k] \le \Lambda |\xi |^2 |\eta |^{2k} \end{aligned}$$

for all $\xi \in \mathbb R^N,$ $\eta \in \mathbb R^n,$ where $0< \lambda \le \Lambda < \infty .$ Here we write $\eta ^k = \eta \, \otimes \cdots \otimes \, \eta$ to denote the *k*-fold tensor product and identify elements $\xi \otimes \eta ^{k} \in \mathbb M_k$ to send $(x_1,\dots ,x_k) \rightarrow \xi \sum _{|\alpha |=k} x^{\alpha }\eta ^{\alpha }.$ These generalise the rank-one matrices from the $k=1$ case.

We will consider the operator

2.26 $$\begin{aligned} \nabla ^k: \mathbb A \nabla ^ku = \sum _{|\alpha |=|\beta |=m} \textrm{D}^{\beta }\left( \mathbb A_{\beta ,\alpha } \textrm{D}^{\alpha }u\right) , \end{aligned}$$

where the coefficients $\mathbb A_{\alpha \beta }$ of $\mathbb A$ satisfies $\xi _1 \cdot \mathbb A_{\alpha ,\beta } \xi _2 = \mathbb A[\xi _1 \otimes \mathrm e^{\alpha },\xi _2 \otimes \mathrm e^{\beta }]$ for all $\xi _1, \xi _2 \in {\mathbb {R}}^N$.

#### Remark 2.7

For our harmonic approximation arguments, we will need the fact that the strict quasiconvexity condition (H2) implies that $\mathbb A[v,w]:= F''(z_0)[v,w]$ is Legendre-Hadamard elliptic. To see this let *F* satisfy Hypotheses 1.1, and for $z_0 \in \mathbb M_k$ and $\xi \in {{\,\textrm{C}\,}}^{\infty }_c(\Omega ,\mathbb R^N)$ consider the functional

2.27 $$\begin{aligned} {\mathcal {J}}(t) = \int _{\Omega } F(z_0+t\nabla ^k\xi ) - F(z_0) - \nu \, \varphi _{1+|z_0|}(t \nabla ^k\xi ) \,\textrm{d}x. \end{aligned}$$

By (H2) we have ${\mathcal {J}}(t) \ge 0$ for all *t*, so it is minimised at $t=0$. Therefore noting that $\varphi _{1+|z_0|}''(0)$ exists and by differentiating under the integral sign, we have ${\mathcal {J}}''(0)$ exists and is non-negative. That is, we have

2.28 $$\begin{aligned} \int _{\Omega } F''(z_0)\nabla ^k\xi : \nabla ^k \xi \,\textrm{d}x \ge \nu \int _{\Omega } \frac{\varphi '(\max \{1,|z_0|\})}{\max \{1,|z_0|\}} |\nabla ^k \xi |^2 \,\textrm{d}x. \end{aligned}$$

From this we deduce that

2.29 $$\begin{aligned} F''(z_0) (\xi \otimes \eta ^k): (\xi \otimes \eta ^k) \ge \frac{\nu \,\varphi '(1)}{1+M} |\xi |^2 |\eta |^{2k} \end{aligned}$$

for all $z_0 \in \mathbb M_k$ with $|z_0|\le M$ and $\xi \in \mathbb R^N, \eta \in \mathbb R^n.$ Note that since we normalised $\varphi (1)=1,$ the ellipticity constant only depends on $\nu , N, \Delta _2(\varphi ).$

#### Proposition 2.8

Let $\varphi \in \Delta _2 \cap \nabla _2$ be an *N*-function and $\mathbb A$ be uniformly Legendre-Hadamard elliptic as above. Then if $\Omega \subset \mathbb R^n$ is a bounded smooth domain, the problem

2.30 $$\begin{aligned} \left\{ \begin{array}{cl} {(-1)^k \nabla ^k: \mathbb A\nabla ^k u}={(-1)^k \nabla ^k:G}& \quad \text { in } {\Omega ,}\\ {\partial _{\nu }^ju}={0}& \quad \text { on }{\partial \Omega , \text { for all } 1 \le j \le k-1,}\\ \end{array}\right. \end{aligned}$$

is uniquely solvable in ${{\,\textrm{W}\,}}_0^{k,\varphi }(\Omega ,\mathbb R^N)$ for all $G \in {{\,\textrm{L}\,}}^{\varphi }(\Omega , \mathbb M_k),$ and the unique solution *u* satisfies

2.31 $$\begin{aligned} \int _{\Omega } \varphi \left( \left|\nabla ^ku\right|\right) \,\textrm{d}x \le C \int _{\Omega } \varphi (|G|) \,\textrm{d}x, \end{aligned}$$

where $$C = C(n,N,k,\Omega ,\lambda ,\Lambda ,\Delta _2(\varphi ),\nabla _2(\varphi ))>0$$.

We will apply this estimate on balls, on which we can note this estimate is scale invariant. Note that the boundary condition (2.30) will be interpreted as simply requiring $u \in {{\,\textrm{W}\,}}^{k,\varphi }_0(\Omega ,\mathbb R^N).$

This result is well-known and follows from the results in Agmon, Douglis, & Nirenberg [5] in the ${{\,\textrm{L}\,}}^p$ setting, which extends to the Orlicz setting by interpolation. We will sketch a more elementary argument based on the interpolation results of Stampacchia [65], using a modern formulation using maximal functions (see e.g.  [25, 50]), which we combine with a Marcinkiewicz-type interpolation argument. In the case $k=1$, these methods were also surveyed by the author in [45, Chapter 3].

#### Sketch of proof

We first establish the result for when $\varphi (t) = t^p,$ for which we let $L = (-1)^k \nabla ^k: \mathbb A\nabla ^k$ viewed as a linear operator ${{\,\textrm{W}\,}}^{k,p}_0(\Omega ,\mathbb R^N) \rightarrow {{\,\textrm{W}\,}}^{-k,p}(\Omega ,\mathbb R^N) \simeq {{\,\textrm{W}\,}}_0^{k,p'}(\Omega ,\mathbb R^N)^*.$ When $p=2$ we can use the Plancherel theorem to show that for any $\omega \subset \mathbb R^n$ open we have

2.32 $$\begin{aligned} \lambda \int _{\omega } |\nabla ^k u |^2 \,\textrm{d}x \le \int _{\omega } \mathbb A[\nabla ^k u, \nabla ^k u] \,\textrm{d}x \end{aligned}$$

for all $u \in {{\,\textrm{W}\,}}^{k,2}_0(\omega ,\mathbb R^N),$ from which we can deduce unique solvability in $\omega$ using the Lax-Milgram lemma. If $p\ge 2$ we will establish an a-priori estimate, so let $u \in {{\,\textrm{C}\,}}^{\infty }_c(\Omega ,\mathbb R^N).$ Then if $x_0 \in {\overline{\Omega }}$ and $0<R<R_0$ with $R_0>0$ sufficiently small, we have the problem

2.33 $$\begin{aligned} \left\{ \begin{array}{ll} {Lw}={Lu}& \quad \text { in }{\Omega \cap \textrm{B}_R(x_0),}\\ {\partial _{\nu }^ju}={0}& \quad \text { on }{\partial \Omega \cap \textrm{B}_R(x_0), \text { for all } 1 \le j \le k-1}\\ \end{array}\right. \end{aligned}$$

admits a unique solution $w \in {{\,\textrm{W}\,}}^{k,2}_0(\Omega \cap \textrm{B}_R(x_0),\mathbb R^N).$ Since the difference $v = u-w$ satisfies $Lv = 0$ in $\Omega \cap \textrm{B}_R(x_0),$ by standard energy methods (see for instance [68, Section 5.11]) we have the uniform estimate2.34By combining these, we will show for all $x_0 \in {\overline{\Omega }}$ and $\theta \in (0,1)$ that

2.35 $$\begin{aligned} {\mathcal {M}}_{\Omega }^{\#}(|\nabla ^ku|)(x_0) \le C \left( \theta \mathcal M_{\Omega }\left( |\nabla ^ku|^2\right) (x_0)^{\frac{1}{2}} + \theta ^{-\frac{n}{2}}{\mathcal {M}}_{\Omega }\left( |G|^2\right) (x_0)^{\frac{1}{2}} \right) , \end{aligned}$$

where we define the localised maximal functions2.362.37for $f \in {{\,\textrm{L}\,}}^1_{{{\,\textrm{loc}\,}}}(\Omega ,\mathbb M_k).$ To see this, for $R<R_0$ note that the above estimates implies that2.38using (2.32) for *w* and (2.34) for *v*. If $R>R_0$ similar estimates hold by a patching argument, from which the pointwise estimate (2.35) follows. Now given $p \ge 2$, taking ${{\,\textrm{L}\,}}^{p/2}$ norms on both sides, we can use the Hardy-Littlewood maximal inequality and the Fefferman-Stein inequality in this setting (see e.g.  [23, 25]) to deduce the estimate

2.39 $$\begin{aligned} \left\Vert \nabla ^ku\right\Vert _{{{\,\textrm{L}\,}}^p(\Omega ,\mathbb M_k)} \le C \left( \theta \left\Vert \nabla ^ku\right\Vert _{{{\,\textrm{L}\,}}^p(\Omega ,\mathbb M_k)} + \theta ^{-\frac{n}{2}} \left\Vert G\right\Vert _{{{\,\textrm{L}\,}}^p(\Omega ,\mathbb M_k)} \right) , \end{aligned}$$

so if $u \in {{\,\textrm{C}\,}}^{\infty }_c(\Omega )$ we deduce ${{\,\textrm{L}\,}}^p$ estimates of the from (2.31) for $p\ge 2.$ By density these estimates extend to all $u \in {{\,\textrm{W}\,}}^{k,p}_0(\Omega ,\mathbb R^N),$ and by regularising $G \in {{\,\textrm{L}\,}}^p(\Omega ,\mathbb M_k)$ and passing the limit using the a-priori estimate we can infer that *L* is an isomorphism ${{\,\textrm{W}\,}}^{k,p}_0(\Omega ,\mathbb R^N) \rightarrow {{\,\textrm{W}\,}}^{-k,p}(\Omega ,\mathbb R^N)$ (injectivity follows form the $p=2$ case). Hence by duality (noting the adjoint operator $L^*$ is an isomorphism) unique solvability extends to the $1<p<2$ range.

For the general Orlicz setting, we will extend the a-priori estimate (2.31), proven for $\varphi (t) = t^p$, to hold for a general *N*-function $\varphi \in \Delta _2 \cap \nabla _2$. This will involve a generalisation of the Marcinkiewicz interpolation theorem, which is proven in [70, Theorem 2] (see also [60]); here one requires that there exists $1< p_0< p_1 < \infty$ and $C>0$ such that

2.40 $$\begin{aligned} \int _t^{\infty } s^{-p_1} \varphi (s) \frac{\textrm{d}s}{s} \le C t^{-p_1} \varphi (t), \quad \int _0^{t} s^{-p_0} \varphi (s) \frac{\textrm{d}s}{s} \le C t^{-p_0} \varphi (t) \end{aligned}$$

for all $t \ge 0$. For general $\varphi \in \Delta _2\cap \nabla _2$, it follows from [51, Theorems 11.7, 11.8] that such $p_0,p_1$ exist, thereby establishing (2.31). Now we can show that $L :{{\,\textrm{W}\,}}^{k,\varphi }_0(\Omega ;{\mathbb {R}}^N) \rightarrow {{\,\textrm{W}\,}}^{-k,\varphi }_0(\Omega ;{\mathbb {R}}^N)$ is an isomorphism by the same argument as in the ${{\,\textrm{L}\,}}^p$ case, noting that uniqueness follows from the inclusion ${{\,\textrm{W}\,}}^{k,\varphi }_0(\Omega ) \subset {{\,\textrm{W}\,}}^{k,p_0}_0(\Omega )$. $\square$

We will also need solvability results for when the right-hand side *g* lies in ${{\,\textrm{W}\,}}^{k-1,\varphi }(\Omega ,\mathbb M_{k-1}).$ This will follow from the above in conjunction with the following (non-optimal) representation theorem.

#### Lemma 2.9

Suppose $\varphi \in \Delta _2 \cap \nabla _2,$ $\ell \ge 0$ and $g \in {{\,\textrm{L}\,}}^{\varphi }(\textrm{B}_R(x_0),\mathbb M_{\ell }),$ where $\textrm{B}_R(x_0)\subset \mathbb R^n$ is any ball. Then there exists $G \in {{\,\textrm{L}\,}}^{\varphi ^{\frac{n}{n-1}}}(\textrm{B}_R(x_0),\mathbb M_{\ell +1})$ such that $-\nabla \cdot G = g$ in $\textrm{B}_R(x_0),$ and we have the corresponding estimate2.41

#### Sketch of proof

We will consider the Newtonian potential (see e.g.  [35, Section 7])

2.42 $$\begin{aligned} G(x) = \frac{-1}{n\omega _n}\int _{\textrm{B}_R(x_0)} \frac{g(y) \otimes (x-y)}{|x- y|^{n}} \,\textrm{d}y, \end{aligned}$$

which satisfies $-\nabla \cdot G = g$ in $\textrm{B}_R(x_0)$ and we claim we have the singular integral estimates

2.43 $$\begin{aligned} \int _{\textrm{B}_R(x_0)} \varphi (|\nabla G|) \,\textrm{d}x \le \int _{\textrm{B}_R(x_0)} \varphi (|g|) \,\textrm{d}x. \end{aligned}$$

Indeed the case $\varphi (t) = t^p$ is proven in [35, Theorem 9.9] (applied to $\mathbb {1}_{\textrm{B}_R(x_0)}g$), and we can extend to general $\varphi \in \Delta _2 \cap \nabla _2$ using the results of [70] analogously as in the above proof. We now apply Poincaré-Sobolev inequality (Lemma 2.3), which gives an extra term arising form the average; this can be bounded using the estimate

2.44 $$\begin{aligned} \begin{aligned} \int _{\textrm{B}_R(x_0)} \varphi (|G|) \,\textrm{d}x&\le \frac{C}{R} \int _{\textrm{B}_R(x_0)} \int _{\textrm{B}_R(x_0)} \varphi (R|g(y)|) |x- y|^{1-n} \,\textrm{d}x \,\textrm{d}y \\&\le C \int _{\textrm{B}_R(x_0)} \varphi (|R g(y)|) \,\textrm{d}y. \end{aligned} \end{aligned}$$

using (2.42), Jensen’s inequality (applied to the measure $\textrm{d}\mu = \frac{C}{R}|x- y|^{1-n} \,\textrm{d}y$) and Fubini’s theorem. $\square$

### A Lipschitz truncation lemma

We will establish the following higher-order version of the Lipschitz truncation lemma of Acerbi & Fusco [1, 2], which we will need for the harmonic approximation argument. The higher order case requires a more delicate extension argument, and was established in Friesecke, James, & Müller [32, Proposition A.2] for $k=2$ using extension results in Ziemer [69]. These arguments straightforwardly generalise to the case of general *k*, which we record here.

#### Proposition 2.10

Let $\varphi \in \Delta _2 \cap \nabla _2$ and $q \in {{\,\textrm{W}\,}}^{k,\varphi }_0(\textrm{B}_R(x_0),\mathbb R^N)$ with $\textrm{B}_R(x_0) \subset \mathbb R^n$ a ball. Then for each $\lambda >0$ there exists $q_{\lambda } \in {{\,\textrm{W}\,}}^{k,\infty }_0(\textrm{B}_R(x_0),\mathbb R^N)$ satisfying the following estimates.

2.45 $$\begin{aligned} \left\Vert \nabla ^kq_{\lambda }\right\Vert _{{{\,\textrm{L}\,}}^{\infty }(\textrm{B}_R(x_0),\mathbb M_k)}&\le C_1\lambda , \end{aligned}$$

2.46 $$\begin{aligned} \int _{\textrm{B}_R(x_0)} \varphi (|\nabla ^kq_{\lambda }|) \,\textrm{d}x&\le C_2\int _{\textrm{B}_R(x_0)} \varphi (|\nabla ^kq|)\,\textrm{d}x, \end{aligned}$$

2.47 $$\begin{aligned} \varphi (\lambda ) {\mathcal {L}}^n\left( \left\{ x \in \textrm{B}_R(x_0) : q(x) \ne q_{\lambda }(x)\right\} \right)&\le C_2\int _{\textrm{B}_R(x_0)} \varphi (|\nabla ^kq|)\,\textrm{d}x, \end{aligned}$$

where $$C_1 = C_1(n,N,k)$$ and $$C_2 = C_2(n,N,k,\Delta _2(\varphi ),\nabla _2(\varphi )).$$

#### Proof

Working componentwise we can assume *q* is scalar-valued, and extending by zero we will also view $q \in {{\,\textrm{W}\,}}^{k,\varphi }(\mathbb R^n)$. and by translation and rescaling we will work with the unit ball $\textrm{B}= \textrm{B}_1(0)$ centred at the origin. We will also work with the precise representatives of $q, \nabla q, \dots , \nabla ^kq,$ and let $L_{\textrm{B}}$ denote the set of Lebesgue points for every $\nabla ^jq$, $0\le j \le k$. Then for $\lambda >0$ consider

2.48 $$\begin{aligned} H_{\lambda } = \left\{ x \in L_{\textrm{B}}: {\mathcal {M}}(|\textrm{D}^{\beta } q|) \le \lambda \text { for all } |\beta |\le k \right\} , \end{aligned}$$

where ${\mathcal {M}} = {\mathcal {M}}_{{\mathbb {R}}^n}$ is the maximal operator defined as in (2.36). Note by boundedness of the maximal operator (using [46, Theorem 1.2.1] noting $\varphi \in \Delta _2 \cap \nabla _2$) we have

2.49 $$\begin{aligned} \int _{{\mathbb {R}}^n} \varphi ({\mathcal {M}}(|\nabla ^jq|)) \,\textrm{d}x \le C\int _{\textrm{B}} \varphi (|\nabla ^jq|) \,\textrm{d}x \end{aligned}$$

for each $0 \le j \le k$, from which it follows that $\mathcal L^n({\mathbb {R}}^n {\setminus } H_{\lambda }) < \infty$. If $\mathcal L^n({\mathbb {R}}^n {\setminus } H_{\lambda }) = 0$ then we can take $q_{\lambda } = q$, otherwise there exists a closed set $A_{\lambda } \subset H_{\lambda }$ for which ${\mathcal {L}}^n({\mathbb {R}}^n {\setminus } A_{\lambda }) \le 2 {\mathcal {L}}^n({\mathbb {R}}^n {\setminus } H_{\lambda })$. We will now construct a ${{\,\textrm{W}\,}}^{k,\infty }$ map $q_{\lambda }$ satisfying $q=q_{\lambda }$ on $A_{\lambda }$, and show this satisfies all the claimed estimates.

For this, we define for all $x \in A_{\lambda }$

2.50 $$\begin{aligned} P_x(y) = \sum _{|\alpha |\le k-1} \frac{\textrm{D}^{\alpha }q(x)}{\alpha !} (y-x)^{\alpha }, \end{aligned}$$

which by [69, Theorem 3.4.1] satisfies2.51for all $x \in A_{\lambda }$ and $|\beta |\le k-1$. Hence by [69, Theorem 3.5.7] we obtain the pointwise estimate

2.52 $$\begin{aligned} |\textrm{D}^{\beta }q(y) - \textrm{D}^{\beta }P_x(y)|\le C \lambda |x- y|^{k-|\beta |} \end{aligned}$$

for all $x,y \in A_{\lambda }$ and $|\beta |\le k-1.$ For the boundary values we claim that

2.53 $$\begin{aligned} |\textrm{D}^{\beta }q(x)|\le C\lambda {{\,\textrm{dist}\,}}(x,\partial \textrm{B})^{k-|\beta |} \end{aligned}$$

holds for all $x \in A_{\lambda }$ and $|\beta |\le k.$ To see this let $z \in \partial \textrm{B}$ such that $|x - z|= d(x) = {{\,\textrm{dist}\,}}(x,\partial \textrm{B}),$ and note that $\textrm{B}_{d(x)}(z) \subset \textrm{B}_{2d(x)}(x).$ Since $\textrm{B}$ is a ball it satisfies an external measure density condition of the from ${\mathcal {L}}^n(\textrm{B}_r(z) {\setminus } \textrm{B}) \ge \nu {\mathcal {L}}^n(\textrm{B}_r(z))$ for all $z \in \partial \textrm{B}$ and $0< r< 1,$ so we have ${\mathcal {L}}^n(\textrm{B}_{2d(x)}(x) {\setminus } \textrm{B}) \ge 2^{-n}\nu {\mathcal {L}}^n(\textrm{B}_{2d(x)}(x)).$ Hence by a suitable Poincaré inequality (applied to the zero extension of *q*, using [69, Corollary 4.5.2]) we have2.54We now combine this with (2.51) and use pointwise bounds for $P_x$, noting $x \in A_{\lambda }$, to estimate2.55thereby establishing (2.53). Now we extend *P* to $A_{\lambda } \cup \partial \textrm{B}$ by setting $P_x = 0$ for all $x \in \partial \textrm{B},$ so by the above estimates we have

2.56 $$\begin{aligned} |\textrm{D}^{\beta }q(x) - \textrm{D}^{\beta }P_x(y)|\le C_1 \lambda |x - y|^{k-|\beta |} \end{aligned}$$

for all $x,y \in A_{\lambda } \cup \partial \textrm{B}$. Hence letting $M > C_1$ to be determined, we define

2.57 $$\begin{aligned} {\widetilde{q}}_{\lambda }(y) = \sup _{x \in A_{\lambda } \cup \partial \textrm{B}} \left( P_{x}(y) - M \lambda |x- y|^{k}\right) , \end{aligned}$$

which by choice of *M* and (2.56) satisfies

2.58 $$\begin{aligned} {\widetilde{q}}_{\lambda }(x) = \left\{ \begin{array}{ll} q(x) &  \text { if } x \in A_{\lambda }, \\ 0 &  \text { if } x \in \partial \textrm{B}. \end{array} \right. \end{aligned}$$

By increasing *M* further if necessary, we claim this satisfies

2.59 $$\begin{aligned} |{\widetilde{q}}_{\lambda }(y) - P_x(y)|\le C\lambda |x- y|^k \end{aligned}$$

for all $x \in A_{\lambda } \cup \partial \textrm{B}$ and $y \in \overline{\textrm{B}}.$ Indeed for $z \in A_{\lambda } \cup \partial \textrm{B}$ we have

2.60 $$\begin{aligned} \begin{aligned}&(P_z(y) - M\lambda |y - z|^k) - P_x(y) \\&\quad \le |P_x(y) - P_z(y)|- M\lambda |y - z|^k \\&\quad \le C\lambda \sum _{j=0}^{k-1} |x - z|^{k-j} \left( |x - y|^j + |z - y|^j\right) - M \lambda |y - z|^k \\&\quad \le C \lambda |y - x|^k + (C - M)\lambda |y - z|^k, \end{aligned} \end{aligned}$$

where we used Young’s inequality and the fact that $|x-z |\le |x - y|+ |y - z|.$ Choosing $M \ge C$ and extremising over *z* implies the estimate (2.59), noting the lower bound follows from the definition.

Now we can apply the extension result [69, Theorem 3.6.2] to deduce the existence of $q_{\lambda } \in {{\,\textrm{W}\,}}^{1,\infty }_0( \textrm{B})$ such that $\left\Vert \nabla ^kq\right\Vert _{{{\,\textrm{L}\,}}^{\infty }( \textrm{B},\mathbb M_k)} \le C\lambda ,$ and such that $q = q_{\lambda }$ on $A_{\lambda }.$

It remains to establish the estimates (2.46), (2.47). For (2.47) we apply Markov’s inequality to estimate

2.61 $$\begin{aligned} \begin{aligned}&\varphi (\lambda ) {\mathcal {L}}^n(\{q \ne q_{\lambda }\}) \le 2\varphi (\lambda ) {\mathcal {L}}^n({\mathbb {R}} \setminus H_{\lambda }) \\&\quad \le C \sum _{j=0}^k\int _{\mathbb R^n} \varphi (\mathcal M(|\nabla ^jq|)) \,\textrm{d}x \le C \sum _{j=0}^k \int _{\textrm{B}} \varphi (|\nabla ^j q|) \,\textrm{d}x \le C \int _{\textrm{B}} \varphi (|\nabla ^kq|) \,\textrm{d}x, \end{aligned}\nonumber \\ \end{aligned}$$

where we have used (2.49) and the Poincaré inequality (Lemma 2.3) in the last two lines. For (2.46) we can estimate

2.62 $$\begin{aligned} \int _{\textrm{B}} \varphi (|\nabla ^kq_{\lambda }|) \,\textrm{d}x \le \int _{\textrm{B}} \varphi (|\nabla ^k q|) \,\textrm{d}x + \int _{\{q \ne q_{\lambda }\}} \varphi \left( \left\Vert \nabla ^k q_{\lambda }\right\Vert _{{{\,\textrm{L}\,}}^{\infty }(\textrm{B},\mathbb M_k)}\right) \,\textrm{d}x, \end{aligned}$$

from which we can conclude noting $\left\Vert \nabla ^k q_{\lambda }\right\Vert _{{{\,\textrm{L}\,}}^{\infty }(\textrm{B},\mathbb M_k)}\le C\lambda$ and applying (2.47). $\square$

## Proof of the regularity theorem

### Caccioppoli inequality of the second kind

We will begin by establishing a Caccioppoli inequality of the second kind in this general growth setting, adapting the estimate of Evans [30]. In the case $k=1$, this argument in the Orlicz setting can be found in [22], and we will show this extends to the case of general *k*.

For this we fix $z_0 \in \mathbb M_k,$ and following [2] we introduce the shifted integrand

3.1 $$\begin{aligned} F_{z_0}(z) = F(z_0+z) - F(z_0) - F'(z_0)z. \end{aligned}$$

Then for each $M \ge 1,$ if $|z_0|\le M$ by distinguishing between the cases when $|z_0 |\le M+1$ and $|z_0 |\ge M+1$ we have

3.2 $$\begin{aligned} |F_{z_0}(z)|&\le C\,\varphi _{1+|z_0 |}(|z|), \end{aligned}$$

3.3 $$\begin{aligned} |F_{z_0}'(z)|&\le C\,\varphi _{1+|z_0 |}'(|z|), \end{aligned}$$

where $C>0$ depends on $n, N,k,K, \Delta _2(\varphi )$ and $G(M):= \sup _{|z|\le 2\,M+1} |F''(z)|.$ Here the second estimate follows from the first by rank-one convexity of $F_{z_0}$ (implied by (H2)).

#### Lemma 3.1

(Caccioppoli inequality) Let $u \in {{\,\textrm{W}\,}}^{k,\varphi }(\Omega ,\mathbb R^N)$ be a minimiser of (1.1), where the integrand *F* satisfies Hypotheses 1.1, and let $M>0.$ Then if $a :\mathbb R^n \rightarrow \mathbb R^N$ is a *k*^th^ order polynomial such that $|\nabla ^k a|\le M,$ there for any $\textrm{B}_R(x_0) \subset \Omega$ we have the estimate3.4where $C = C(n,N,k,K,\nu ,\Delta _2(\varphi ),M,G(M))>0$.

#### Proof

We will suppress the $x_0$ dependence to simplify notation. Fix $0<t<s<R,$ and let $\eta \in {{\,\textrm{C}\,}}^{\infty }_c(\textrm{B}_R)$ be a radial cut-off such that $\mathbb {1}_{\textrm{B}_t} \le \eta \le \mathbb {1}_{\textrm{B}_s}$ and $|\nabla ^j\eta |\le \frac{C}{(s-t)^j}$ for each $1 \le j \le k.$ Given *a*, we set $w = u-a,$ ${\widetilde{F}} = F_{\nabla ^k a}$ and ${\widetilde{\varphi }} = \varphi _{1+|\nabla ^k a|}$, noting that ${\widetilde{\varphi }}\sim \varphi _{1+M}$. Observe that minimality of *u* implies that

3.5 $$\begin{aligned} \int _{\textrm{B}_R} {\widetilde{F}}(\nabla ^k w ) \,\textrm{d}x \le \int _{\textrm{B}_R} {\widetilde{F}}(\nabla ^kw + \nabla ^k\xi ) \,\textrm{d}x \end{aligned}$$

for any $\xi \in {{\,\textrm{W}\,}}^{k,\varphi }_0(\textrm{B}_R)$, using that $\int _{\textrm{B}_R} F'(\nabla ^k a )\nabla ^k\xi \,\textrm{d}x = 0$. Then by the strict quasiconvexity condition (H2) we have

3.6 $$\begin{aligned} \begin{aligned} \nu \int _{\textrm{B}_s} {\widetilde{\varphi }}(|\nabla ^k(\eta w)|) \,\textrm{d}x&\le \int _{\textrm{B}_s} {\widetilde{F}}(\nabla ^k(\eta w)) \,\textrm{d}x \\&\le \int _{\textrm{B}_s} {\widetilde{F}}(\nabla ^k w) \,\textrm{d}x + \int _{\textrm{B}_s} {\widetilde{F}}(\nabla ^k(\eta w)) - {\widetilde{F}}(\nabla ^kw) \,\textrm{d}x. \end{aligned} \end{aligned}$$

Now using the minimising property of *w* and noting $\eta w = w$ on $\textrm{B}_t$,

3.7 $$\begin{aligned} \begin{aligned}&\int _{\textrm{B}_t} {\widetilde{\varphi }}(|\nabla ^k(\eta w)|) \,\textrm{d}x \\&\quad \le \frac{1}{\nu }\int _{\textrm{B}_s} {\widetilde{F}}(\nabla ^k((1-\eta )w)) \,\textrm{d}x + \frac{1}{\nu }\int _{\textrm{B}_s} {\widetilde{F}}(\nabla ^k(\eta w)) - {\widetilde{F}}(\nabla ^k w) \,\textrm{d}x \\&\quad \le C \int _{\textrm{B}_s \setminus \textrm{B}_t} {\widetilde{\varphi }}(|\nabla ^k((1-\eta )w)|) + {\widetilde{\varphi }}(|\nabla ^k (\eta w) |) + {\widetilde{\varphi }}(|\nabla ^k w|) \,\textrm{d}x \\&\quad \le C \int _{\textrm{B}_s \setminus \textrm{B}_t} {\widetilde{\varphi }}(|\nabla ^k w|) \,\textrm{d}x + C \sum _{j=0}^{k-1}\int _{\textrm{B}_s} {\widetilde{\varphi }}\left( \frac{|\nabla ^j w |}{(s-t)^{k-j}} \right) \,\textrm{d}x, \end{aligned} \end{aligned}$$

using the $\Delta _2$-condition. By filling the hole, setting $\theta = \frac{C}{C+1} \in (0,1)$ gives

3.8 $$\begin{aligned} \int _{\textrm{B}_t} {\widetilde{\varphi }}(|\nabla w|) \,\textrm{d}x \le \theta \int _{\textrm{B}_s} {\widetilde{\varphi }}(|\nabla w|) \,\textrm{d}x + C_{*}\sum _{j=0}^{k-1}\int _{\textrm{B}_s} {\widetilde{\varphi }}\left( \frac{|\nabla ^j w |}{(s-t)^{k-j}} \right) \,\textrm{d}x. \end{aligned}$$

Now by the interpolation estimate (Lemma 2.6) we estimate

3.9 $$\begin{aligned} \begin{aligned} C_{*}\sum _{j=0}^{k-1}\int _{\textrm{B}_s} {\widetilde{\varphi }}\left( \frac{|\nabla ^j w |}{(s-t)^{k-j}} \right) \,\textrm{d}x&\le \frac{1-\theta }{2} \int _{\textrm{B}_s} {\widetilde{\varphi }}(|\nabla ^kw|) \,\textrm{d}x + C \int _{\textrm{B}_s} {\widetilde{\varphi }}\left( \frac{|w|}{(s-t)^k} \right) \,\textrm{d}x, \end{aligned}\nonumber \\ \end{aligned}$$

allowing us to absorb the intermediate terms. Finally by an iteration argument (adapting [22, Lemma 3.1]) we deduce that

3.10 $$\begin{aligned} \int _{\textrm{B}_{R/2}} {\widetilde{\varphi }}(|\nabla ^k w|) \,\textrm{d}x \le C \int _{\textrm{B}_R} {\widetilde{\varphi }}\left( \frac{|w|}{R^k} \right) \,\textrm{d}x, \end{aligned}$$

as required. $$\square $$

### Harmonic approximation

Our second ingredient will involve approximation by solutions *h* to the linearised equation, adapting a recent strategy of Gmeineder & Kristensen [39], which has also been applied for instance in [7, 31, 38, 44]. A key feature of this argument is that we only use the Legendre-Hadamard ellipticity condition (2.29) derived in Remark 2.7 and that *u* is *F*-extremal, so it can be applied more generally to infer regularity in situations where a suitable Caccioppoli inequality holds. This was exploited by the author in [44], and we will also use this observation in Sect. 4 for the case of strong local minima.

We will also need some additional estimates on the integrand *F*. Considering the shifted integrand $F_{z_0}$ defined in (3.1), where $z_0 \in \mathbb M_k$ with $|z_0|\le M,$ we recall that $F_{z_0}''(0) = F''(z_0)$ satisfies the Legendre-Hadamard ellipticity bound (2.29) by Remark 2.7. Further we also need the perturbation estimate

3.11 $$\begin{aligned} |F_{z_0}'(z) - F_{z_0}''(0)z|\le C \omega _M(|z|)\left( |z |+ \varphi _{|z_0 |}'(|z|)\right) . \end{aligned}$$

Here $\omega _M$ denotes the modulus of continuity of $F''$ on $\{|z|\le 2M\},$ that is $\omega _M$ is a non-negative non-decreasing continuous concave function $[0,\infty ) \rightarrow [0,1]$ satisfying $\omega _M(0)=0$ and

3.12 $$\begin{aligned} |F''(z) - F''(w)|\le 2G(M) \omega _M(|z-w|) \end{aligned}$$

for all $z,w \in \mathbb M_k$ such that $|z|,|w|\le 2\,M+1.$ The claimed estimate can be obtained by combining the above with the growth bound (3.3).

#### Lemma 3.2

(Harmonic approximation) Let $u \in {{\,\textrm{W}\,}}^{k,\varphi }(\Omega ,\mathbb R^N)$ be *F*-extremal, where *F* satisfies Hypotheses 1.1, and let $M>0,$ $\delta \in (0,1).$ Then if $a :\mathbb R^n \rightarrow \mathbb R^N$ is a *k*^th^ order polynomial such that $|\nabla ^k a|\le M,$ for any $\textrm{B}_R(x_0) \subset \Omega$ the Dirichlet problem

3.13 $$\begin{aligned} \left\{ \begin{array}{ll} {(-1)^k \nabla ^k: F''(\nabla ^k a)\nabla ^k h}={0}& \quad \text { in }{\textrm{B}_R(x_0),}\\ \qquad \qquad \qquad {\partial _{\nu }^jh}={\partial _{\nu }^j(u-a)}& \quad \text { on }{\partial \textrm{B}_R(x_0),\ \ 1 \le j \le k-1,}\\ \end{array}\right. \end{aligned}$$

admits a unique solution $h \in (u-a)+{{\,\textrm{W}\,}}^{k,\varphi }_0(\Omega ,\mathbb R^N),$ which satisfies the estimates3.14and3.15Here $\gamma _M: [0,\infty ) \rightarrow [0,\infty )$ is a non-decreasing continuous function such that $\gamma _M(0)=0$ and we have $C_{\delta } = C(n,N,k,K,\nu ,\Delta _2(\varphi ),\nabla _2(\varphi ),M,G(M),\delta )>0.$

#### Proof

Suppressing the $x_0$-dependence, set $w=u-a,$ ${\widetilde{F}} = F_{\nabla ^k a},$ and ${\widetilde{\varphi }} = \varphi _{1+|\nabla ^k a|}$ noting that ${\widetilde{\varphi }} \sim \varphi _{1+M}.$ We know by Proposition 2.8 that a unique solution *h* exists and satisfies the modular estimate (3.14), and since *w* is $\widetilde{F}$-extremal, using the respective weak formulations (1.2), (3.13) we have

3.16 $$\begin{aligned} \int _{\textrm{B}_R} {\widetilde{F}}''(0)\nabla ^k(w-h): \nabla ^k q \,\textrm{d}x = \int _{\textrm{B}_R} ({\widetilde{F}}''(0)\nabla ^k w - {\widetilde{F}}'(\nabla ^k w)): \nabla ^k q \,\textrm{d}x, \end{aligned}$$

for $q \in {{\,\textrm{W}\,}}^{k,\infty }_0(\textrm{B}_R,\mathbb R^N).$ Following [39] we wish to choose our test function so we obtain ${\widetilde{\varphi }}(R^{-1}|\nabla ^{k-1}( w- h)|)$ on the left-hand side, so to achieve this we choose *q* to be the solution of the dual problem

3.17 $$\begin{aligned} \left\{ \begin{array}{cl} {(-1)^k \nabla ^k: F''(\nabla ^k a)\nabla ^k q}={(-1)^{k-1}\nabla ^{k-1}: g}& \quad \text { in }{\textrm{B}_R,}\\ \quad {\partial _{\nu }^jq}={0}& \quad \text { on } {\partial \textrm{B}_R, \ \ 0 \le j \le k-1,}\\ \end{array}\right. \end{aligned}$$

where

3.18 $$\begin{aligned} g = {\widetilde{\varphi }}\left( \frac{|\nabla ^{k-1}(w- h)|}{R} \right) \frac{\nabla ^{k-1}(w-h)}{|\nabla ^{k-1}(w-h)|^2}. \end{aligned}$$

Since $t \mapsto \frac{{\widetilde{\varphi }}(t)}{t}$ is an *N*-function satisfying ${\widetilde{\varphi }}^*({\widetilde{\varphi }}(t)/t) \sim {\widetilde{\varphi }}(t)$ uniformly in *t*,  we have *g* is well-defined and satisfies the estimate3.19Hence by Lemma 2.9 we can write $g = - \nabla \cdot G$ with $G \in {{\,\textrm{L}\,}}^{\varphi ^{\frac{n}{n-1}}}(\textrm{B}_R,\mathbb M_k),$ so then Proposition 2.8 gives the existence of a unique $q \in {{\,\textrm{W}\,}}^{k,\varphi ^{\frac{n}{n-1}}}_0(\textrm{B}_R,\mathbb R^N)$ and a corresponding modular estimate, which we combine with (3.19) to obtain3.20In general we cannot use *q* in (3.16) since it may not lie in ${{\,\textrm{W}\,}}^{k,\infty }(\textrm{B}_R,{\mathbb {R}}^N)$, so we will need to take a higher order Lipschitz truncation. Letting $\lambda >0$ to be determined, applying Proposition 2.10 with the *N*-function $({\widetilde{\varphi }}^*)^{\frac{n}{n-1}}$ gives $q_{\lambda } \in {{\,\textrm{W}\,}}^{k,\infty }(\textrm{B}_R,\mathbb R^N)$ satisfying $\left\Vert \nabla ^kq_{\lambda }\right\Vert _{{{\,\textrm{L}\,}}^{\infty }(\textrm{B}_R,\mathbb M_k)} \le C\lambda$ and3.213.22For this choice of $q_{\lambda }$ we have for each $\delta \in (0,1),$where we have used the perturbation estimate (3.11) and Young’s inequality (2.7). It remains to estimate each of these terms separately; for the first inequality we use the fact that ${\widetilde{\varphi }}^*({\widetilde{\varphi }}'(t)) \le C\varphi (t)$ to estimate3.23and for the second term we use the fact that $\omega _M \le 1$ and apply Jensen’s inequality to the concave function $\omega _M \circ {\widetilde{\varphi }}^{-1}$ to estimate3.24For the third term we apply Hölder’s inequality along with the Lipschitz truncation estimates (3.21), (3.22) to estimate3.25We can now choose $\lambda >0$ sufficiently large so the third term can be absorbed to the left-hand side; for this we can take $\lambda$ to satisfy3.26Now using the doubling property for ${\widetilde{\varphi }}, {\widetilde{\varphi }}^*$ and writing $\vartheta _M(t) = t + {\widetilde{\varphi }} \circ ({\widetilde{\varphi }}^*)^{-1}(t)$ we get3.27Finally note that since $w-h \in {{\,\textrm{W}\,}}^{k,\varphi }_0(\textrm{B}_R,\mathbb R^N),$ by the Poincaré inequality and (3.14) we have3.28so setting $\gamma _M(t) = \frac{\vartheta _M(t)}{t}\, \omega _M \circ {\widetilde{\varphi }}^{-1}(t)$ the result follows. $\square$

### Excess decay estimate

We now combine the above estimates to conclude, which will involve establishing estimates for the *excess energies* defined for $M>0$ as3.29

#### Lemma 3.3

(Excess decay estimate) Let $u \in {{\,\textrm{W}\,}}^{k,\varphi }(\Omega ,\mathbb R^N)$ be a minimiser of (1.1) where the integrand *F* satisfies Hypotheses 1.1, and let $M>0,$ $\delta >0.$ Then for all $\sigma \in \left( 0,\frac{1}{4}\right)$ and $\delta \in (0,1),$ letting $\gamma _M$ as in Lemma 3.2, if $\textrm{B}_R(x_0)\subset \Omega$ such that $|(\nabla ^ku)_{\textrm{B}_R(x_0})|\le M$ and $\mathrm E_M(x_0,R)\le 1$ we have

3.30 $$\begin{aligned} \mathrm E_M(x_0,\sigma R) \le C \left( \sigma + C_{\sigma }\delta + C_{\sigma ,\delta }\gamma _M(\mathrm E_M(x_0,R)) \right) \mathrm E_M(x_0,R), \end{aligned}$$

where the constants depend also on $n, N, k,K,\nu \Delta _2(\varphi ),\nabla _2(\varphi ), M$ and *G*(*M*).

#### Proof

We let $a_1: \mathbb R^n \rightarrow \mathbb R^N$ be a *k*^th^ order polynomial satisfying3.31for all $|\alpha |\le k$ and apply Lemma 3.2 on $\textrm{B}_R(x_0)$ with $a_1,$ obtaining the unique solution *h* to

3.32 $$\begin{aligned} \left\{ \begin{array}{cl} {(-1)^k \nabla ^k: F''(\nabla ^k a_1)\nabla ^kh}={0}& \quad \text { in }{\textrm{B}_R}\\ \qquad \qquad \qquad \qquad {\partial _{\nu }^jh}={\partial _{\nu }^j(u-a_1)}& \quad \text { on }{\partial \textrm{B}_R,\ \ 0 \le j \le k-1.}\\ \end{array}\right. \end{aligned}$$

Combining the Poincaré inequality (Remark 2.4) with the approximation estimate (3.15) from Lemma 3.2 we have

3.33 $$\begin{aligned} \begin{aligned}&\int _{\textrm{B}_R(x_0)} \varphi _{1+M}\left( \frac{|u - a_1-h|}{R^k} \right) \,\textrm{d}x \\&\quad \le \int _{\textrm{B}_R(x_0)} \varphi _{1+M}\left( \frac{|\nabla ^{k-1}(u - a_1-h)|}{R} \right) \,\textrm{d}x \\&\quad \le \left( \delta + C_{\delta }\gamma _M\left( \mathrm E_M(x_0,R) \right) \right) \mathrm E_M(x_0,R). \end{aligned} \end{aligned}$$

Now let $a_2(x) = \sum _{|\alpha |\le k} \frac{\textrm{D}^{\alpha }h(x_0)}{\alpha !} (x-x_0)^{\alpha },$ and put $a=a_1+a_2.$ This satisfies $\varphi _{1+M}(|\nabla ^ka_2|) \le C(M+1),$ so $|\nabla ^ka|\le C(M)$ and hence $\varphi _{1+|\nabla ^ka|} \sim \varphi _{1+M}.$ Therefore applying the Caccioppoli inequality (Lemma 3.1) with *a* on $\textrm{B}_{2\sigma R}(x_0)$ we obtain3.34Now as *h* is $F''(\nabla ^ka)$-harmonic, by interior regularity estimates (see for instance [68, Section 5.11]) and (3.14) we have

3.35 $$\begin{aligned} \sup _{\textrm{B}_{2\sigma R}} \frac{|h(x) - a_2(x)|}{(2\sigma R)^k} \le \frac{2\sigma R}{(k+1)!} \sup _{\textrm{B}_{R/2}(x_0)} |\nabla ^{k+1}h|\le C\varphi _{1+M}^{-1}\left( \sigma \mathrm E_M(x,R)\right) , \end{aligned}$$

so together with (3.33) we obtain

3.36 $$\begin{aligned} \begin{aligned} \mathrm E_M(x_0,\sigma R) \le C_{\sigma } \left( \delta + C_{\delta } \gamma _M(\mathrm E_M(x_0,R)) \right) \mathrm E_M(x_0,R) + C \sigma \mathrm E_M(x_0,R), \end{aligned} \end{aligned}$$

which is the desired estimate. $\square$

We can now conclude in the usual way.

#### Proof of Theorem 1.2

Suppose $\textrm{B}_R(x_0) \subset \Omega$ such that $|(\nabla ^ku)_{\textrm{B}_R(x_0)}|\le M,$ and let $x \in \textrm{B}_{R/2}(x_0).$ Then since $|(\nabla ^ku)_{\textrm{B}_{R/2}(x)}|\le 2^nM$ and $\mathrm E_M(x,R/2) \le 2^n\varepsilon \le 1$ shrinking $\varepsilon$ if necessary, we can apply Lemma 3.3 above with *M* replaced by $2^nM$ obtain

3.37 $$\begin{aligned} \mathrm E_M(x,\sigma R/2) \le C\left( \sigma + C_{\sigma }\delta + C_{\sigma ,\delta } \gamma _M(\varepsilon ) \right) \mathrm E_M(x,R/2), \end{aligned}$$

for $\sigma \in \left( 0,\frac{1}{4} \right)$ and $\delta >0.$ We now choose $\sigma >0$ sufficiently small to ensure $C\sigma < \frac{1}{3}\sigma ^{2\alpha }$ and $\delta >0$ small enough so $CC_{\sigma }\delta < \frac{1}{3}\sigma ^{2\alpha },$ and finally $\varepsilon >0$ so that $CC_{\sigma ,\delta }\gamma _M(\varepsilon ) < \frac{1}{3}\sigma ^{2\alpha },$ which gives

3.38 $$\begin{aligned} \mathrm E_M(x,\sigma R/2) \le \sigma ^{2\alpha }\mathrm E_M(x,R/2). \end{aligned}$$

We claim, by iterating the above, that we have

3.39 $$\begin{aligned} |(\nabla ^k u)_{\textrm{B}_{\sigma ^{j-1}R/2}(x)}|&\le 2^{n+1}M, \end{aligned}$$

3.40 $$\begin{aligned} \mathrm E_M(x,\sigma ^jR/2)&\le \sigma ^{2j\alpha } \mathrm E_M(x,R/2) \end{aligned}$$

for all $j \ge 1,$ provided $\varepsilon >0$ is sufficiently small. Indeed proceeding inductively, if this holds for for all $1 \le i \le j,$ then note first that for such *i* we use Jensen’s inequality to bound3.41Hence by shrinking $\varepsilon >0$ further if necessary to ensure that $\varphi _{1+M}^{-1}(\delta \varepsilon ) \le C\sqrt{\delta }$ for all $\delta \in (0,1),$ we can estimate

3.42 $$\begin{aligned} \begin{aligned} |(\nabla ^k u)_{\textrm{B}_{\sigma ^{j}R/2}(x)}|&\le 2^nM + \sum _{i=1}^{j} |(\nabla ^k u)_{\textrm{B}_{\sigma ^iR/2}(x)} - (\nabla ^ku)_{\textrm{B}_{\sigma ^{i-1}}(x)}|\\&\le 2^nM + \sum _{i=0}^{j-1} \varphi _{1+M}^{-1}(\sigma ^{2i\alpha }\varepsilon ) \le 2^nM + \varepsilon \sum _{i=0}^{\infty } \sigma ^{i\alpha }, \end{aligned} \end{aligned}$$

which can be chosen to be less than $2^{n+1}M$ if $\varepsilon >0$ is sufficiently small, establishing (3.39). Therefore we can iteratively apply the excess decay estimate with the same parameters to deduce (3.40). Hence for any $r \in (0,R/2)$ we have $E(x,r) \le \varphi _{1+M}(2^{n+1}\varepsilon ) \le 1$ and so3.43Since this holds for all $x \in \textrm{B}_{R/2}(x)$ and $0< r < R/2,$ by the Campanato-Meyers characterisation of Hölder continuity (see for instance [36, Theorem 2.9]) it follows that $\nabla ^k u$ is ${{\,\textrm{C}\,}}^{0,\alpha }$ in $\textrm{B}_{R/2}(x_0).$ $\square$

## Extension to strong local minimisers

We will conclude our discussion with a straightforward extension of our main theorem to the setting of ${{\,\textrm{W}\,}}^{k,\psi }$-local minimisers. To be more precise, we consider the following.

### Definition 4.1

Let *F* satisfy Hypotheses 1.1, and $\psi$ be an *N*-function. Then we say $u \in {{\,\textrm{W}\,}}^{k,\varphi }(\Omega ,\mathbb R^N)$ is a *strong* ${{\,\textrm{W}\,}}^{k,\psi }$-*local minimiser* if there exists $\delta >0$ such that if $\xi \in {{\,\textrm{W}\,}}^{k,\psi }_0(\Omega ,\mathbb R^N)$ with $\int _{\Omega } \psi (|\nabla ^k \xi |) \,\textrm{d}x < \delta ,$ then we have

4.1 $$\begin{aligned} {\mathcal {F}}(u) \le {\mathcal {F}}(u+\xi ). \end{aligned}$$

We also say *u* is a ${{\,\textrm{W}\,}}^{k,\infty }$-*local minimiser* if (4.1) is satisfied whenever $\xi \in {{\,\textrm{W}\,}}^{k,\infty }_0(\Omega ,\mathbb R^N)$ such that $\left\Vert \nabla ^k\xi \right\Vert _{{{\,\textrm{L}\,}}^{\infty }(\Omega ,\mathbb M_k)} < \delta .$

The regularity of strong ${{\,\textrm{W}\,}}^{1,q}$-minimisers was first considered by Kristensen & Taheri [48], who established a partial regularity theorem in the case of superquadratic ($p\ge 2$) growth. These results have been extended for instance in [11, 12, 14, 62].

### Theorem 4.2

($\varepsilon$-regularity theorem for local minimisers) Let *F* satisfy Hypotheses 1.1, $\Omega \subset {\mathbb {R}}^n$ be a bounded domain and let $u \in {{\,\textrm{W}\,}}^{k,\varphi }(\Omega ,\mathbb R^N)$ satisfy one of the following minimality properties:

(i) There is an *N*-function $\psi$ such that *u* is a strong ${{\,\textrm{W}\,}}^{k,\psi }$-minimiser, and we have
  4.2 $$\begin{aligned} \int _{\Omega '} \psi (\lambda |\nabla ^ku|) \,\textrm{d}x < \infty \end{aligned}$$
  for all $\lambda >0$ and $\Omega ' \Subset \Omega$.
(ii) We have *u* is a ${{\,\textrm{W}\,}}^{k,\infty }$-minimiser and moreover $u \in {{\,\textrm{W}\,}}^{k,\infty }_{\textrm{loc}}(\Omega ,{\mathbb {R}}^N)$ such that
  4.3 $$\begin{aligned} \limsup _{r \rightarrow 0} \left( \mathop {\mathrm {ess\ sup}}\limits _{y \in \textrm{B}_r(x)} \, |\nabla ^k u -(\nabla ^k u)_{\textrm{B}_r(x)}|\right) < \delta \end{aligned}$$
  locally uniformly in $x \in \Omega$.

Then for each $x_0 \in \Omega ,$ $M>0$ and $\alpha \in (0,1),$ there exists $\varepsilon >0$ and $R_0>0$ such that for any $R<R_0$ for which $|(\nabla ^ku)_{\textrm{B}_R(x_0)}|\le M$ and4.4we have *u* is of class ${{\,\textrm{C}\,}}^{k,\alpha }$ in $\textrm{B}_{R/2}(x_0).$

### Remark 4.3

If $\psi$ satisfies the $\Delta _2$ condition then (4.2) is equivalent to requiring that $u \in {{\,\textrm{W}\,}}^{k,\psi }_{{{\,\textrm{loc}\,}}}(\Omega ,\mathbb R^N),$ however extra care is needed in the absence of the $\Delta _2$-condition due the failure of suitable density results in ${{\,\textrm{L}\,}}^{\psi }.$ To the best of our knowledge is not known if this additional condition (4.2) is necessary, even in the polynomial case when $\psi = t^q.$

In the ${{\,\textrm{W}\,}}^{k,\infty }$ case however, the construction in [48, Section 7] implies that it is insufficient to assume that *u* is a ${{\,\textrm{W}\,}}^{k,\infty }$-local minimiser that lies in ${{\,\textrm{W}\,}}^{k,\infty }_{{{\,\textrm{loc}\,}}}(\Omega ,\mathbb R^N).$

The key difference from the minimising setting is the lack of a full Caccioppoli inequality; to apply the minimising condition we need to work on balls of sufficiently small radii, which prevents us from applying the iteration argument used in the proof of Lemma 3.1. However one can still establish a weaker form which will suffice to complete the argument.

### Lemma 4.4

(Caccioppoli-type inequality) Assume the setup of Theorem 4.2, and let $\kappa \in (0,1).$ Then there is $R_0 > 0$ and $\varepsilon >0$ such that if $\textrm{B}_r(x) \subset \textrm{B}_{R_0}(x_0) \Subset \Omega$ for which $|(\nabla ^ku)_{\textrm{B}_r(x)}|\le M$ and $\mathrm E_M(x,r) \le \varepsilon ,$ then define $a_{x,r}$ such that $\nabla ^{k-1}a_{x,r}$ satisfies Lemma 2.5 in $\textrm{B}_r(x)$ with $\nabla ^{k-1}u$ and that4.5for all $|\alpha |\le k-2.$ Then we have4.6

### Proof

Fix $\zeta \in (0,1)$ to be specified later, and let $0<t<s<r$ such that $r \le \zeta (s-t).$ Then let $\eta \in {{\,\textrm{C}\,}}^{\infty }_c(\textrm{B}_R(x_0))$ be a cutoff such that $\mathbb {1}_{\textrm{B}_t(x)} \le \eta \le \mathbb {1}_{\textrm{B}_s(x)}$ and $|\nabla ^j\eta |\le \frac{C}{(s-t)^{j}}$ for all $0 \le j \le k.$ We wish to use the minimising condition with $\xi = \eta (u-a_{x,r}),$ so we need to verify this is possible provided $R_0$ is sufficiently small.

In the case of (i), we we will use the Poincaré inequality proved in [8, Lemma 1] which is valid for general *N*-functions $\psi$. We claim this allows us to estimate

4.7 $$\begin{aligned} \begin{aligned} \int _{\textrm{B}_r(x)} \psi (|\nabla \xi |) \,\textrm{d}y&\le \sum _{j=0}^k \int _{\textrm{B}_r(x)} \psi \left( C\,\frac{|\nabla ^j (u-a_{x,r})|}{(s-t)^{k-j}} \right) \,\textrm{d}y \\&\le C\sum _{j=0}^{k} \int _{\textrm{B}_r(x)} \psi \left( \frac{Cr^{k-j}}{(s-t)^{k-j}}|\nabla ^{k} (u-a_{x,r})|\right) \,\textrm{d}y \\&\le C \int _{\textrm{B}_{R_0}(x_0)} \psi \left( C \zeta ^{-k} (|\nabla ^ku|+M) \right) \,\textrm{d}y, \end{aligned} \end{aligned}$$

using the additivity inequality $\psi (s+t) \le \psi (2s)+\psi (2t)$ in the first line, followed by iteratively applying the Poincaré inequality using (4.5) which applies for all $|\alpha |\le k-1$ by choice of $\nabla ^{k-1}a_{x,r}.$ Then we can choose $R_0>0$ to be sufficiently small so this bound is smaller than $\delta$, thereby ensuring that $\xi$ is a valid test function. In the case of (ii) we can estimate

4.8 $$\begin{aligned} \begin{aligned} \left\Vert \nabla ^k\xi \right\Vert _{{{\,\textrm{L}\,}}^{\infty }(\Omega ,\mathbb M_k)}&\le \left\Vert \nabla ^ku - (\nabla ^ku)_{\textrm{B}_R(x_0)}\right\Vert _{{{\,\textrm{L}\,}}^{\infty }(\textrm{B}_r(x),\mathbb M_k)} +|\nabla ^ka_{x,r} - (\nabla ^ku)_{\textrm{B}_R(x_0)}|\\&\quad + C\sum _{j=0}^{k-1} \left\Vert \frac{\nabla ^j(u-a_{x,r})}{(s-t)^{k-j}}\right\Vert _{{{\,\textrm{L}\,}}^{\infty }(\textrm{B}_r(x),\mathbb M_j)} \\&\le \delta _1 + |\nabla ^ka_{x,r} - (\nabla ^ku)_{\textrm{B}_R(x_0)}|\\&\quad +C \sum _{j=0}^{k-1} \zeta ^{j-k} \left\Vert r^{k-j}(\nabla ^j(u-a_{x,r}))\right\Vert _{{{\,\textrm{L}\,}}^{\infty }(\textrm{B}_r(x),\mathbb M_j)}, \end{aligned} \end{aligned}$$

where $R_0$ is chosen to be sufficiently small so that

4.9 $$\begin{aligned} \delta _1:= \mathop {\mathrm {ess\ sup}}\limits _{y \in \textrm{B}_{r}(x)} \, |\nabla ^k u -(\nabla ^k u)_{\textrm{B}_r(x)}|< \delta , \end{aligned}$$

for all $\textrm{B}_r(x) \subset \textrm{B}_R(x_0)$, using the additional condition (4.3). Now an analogous argument as in [48, p. 76] allows us to prove the pointwise estimate

4.10 $$\begin{aligned} \begin{aligned}&\left\Vert r^{k-j}(\nabla ^j(u-a_{x,r}))\right\Vert _{{{\,\textrm{L}\,}}^{\infty }(\textrm{B}_r(x),\mathbb M_j)} \\&\quad \le (2N)^{k-j} \left\Vert \nabla ^k(u - a_{x,r})\right\Vert _{{{\,\textrm{L}\,}}^{\infty }(\textrm{B}_r(x),\mathbb M_k)}, \end{aligned} \end{aligned}$$

and we can also apply (2.19) from Lemma 2.5 to estimate
4.11From this we claim we have the interpolation estimate
4.12for all $\gamma > 0$. We will show the first inequality for general $u \in {{\,\textrm{W}\,}}^{k,\infty }(\textrm{B}_r(x),{\mathbb {R}}^N)$ by means of a contradiction argument, noting the second line follows from Jensen’s inequality. Rescaling to the case of the unit ball $\textrm{B}= \textrm{B}_1(0)$ we will assume the inequality does not hold, so there exists $(u_m)_m \subset {{\,\textrm{W}\,}}^{k,\infty }(\textrm{B},{\mathbb {R}}^N)$ and $0 \le j \le k-1$ for which for which
4.13holds for all *m*, where $a_{m}$ is defined as $a_{0,1}$ for $u_m$; in particular $(\nabla ^i(u_m-a_m))_{\textrm{B}_r(x)} = 0$ for all $0\le i \le k-1$ and (4.11) holds. Then by (4.10) and Arzelà-Ascoli, passing to a subsequence there exists *v* such that $\nabla ^i(u_m - a_{x,r,m}) \rightarrow \nabla ^iv$ uniformly on $\textrm{B}$ for all $0 \le i \le k-1$, and since , combining with (4.11) we see that $(\nabla ^kv)_{\textrm{B}} = 0$. Therefore since $\nabla ^kv \equiv 0$ in $\textrm{B}$ and $(\nabla ^iv)_{\textrm{B}} = 0$ for all $0 \le i \le N$, it follows that $v \equiv 0$ in $\textrm{B}$, contradicting the fact that $\Vert \nabla ^j(u_m - a_m)\Vert _{{{\,\textrm{L}\,}}^{\infty }(\textrm{B},{\mathbb {M}}_j)}=1$ for all *m*. Hence (4.12) holds.

Therefore combining our estimates in (4.8) we have

4.14 $$\begin{aligned} \Vert \nabla ^k \xi \Vert _{{{\,\textrm{L}\,}}^{\infty }(\Omega ,{\mathbb {M}}_k)} \le \delta _1 + \gamma \Vert \nabla ^k(u-a_{x,r})\Vert _{{{\,\textrm{L}\,}}^{\infty }(\textrm{B}_R(x),{\mathbb {M}}_k)} + C_{\gamma } \varphi _{1+M}^{-1}(\varepsilon ), \end{aligned}$$

which can be chosen to be smaller than $\delta$ by shrinking $\gamma , \varepsilon >0$ as necessary. Hence, in both cases, the $\xi$ is a valid test function.

Now we can argue as in Lemma 3.1 from the minimising case; writing ${\widetilde{\varphi }} = \varphi _{1+M},$ noting that $w_{x,r} = u - a_{x,r}$ is $F_{\nabla ^ka_{x,r}}$-extremal we can estimate

4.15 $$\begin{aligned} \int _{\textrm{B}_t(x)} {\widetilde{\varphi }}\left( |\nabla ^kw_{x,r}|\right) \,\textrm{d}y \le \theta \int _{\textrm{B}_s(x)} {\widetilde{\varphi }}\left( |\nabla ^kw_{x,r}|\right) \,\textrm{d}y + C \int _{\textrm{B}_r} {\widetilde{\varphi }}\left( \frac{|w_{x,r}|}{(s-t)^k}\right) \,\textrm{d}y. \end{aligned}$$

analogously to (3.7)–(3.9), where $\theta >0$ is given and independent of *s*, *t*. Then there is some $\ell \ge 1$ such that $\theta ^\ell \le \kappa ,$ so we will iteratively apply this estimate $\ell$-times.

For this take $s_j = (1-\frac{j}{2\ell })r, t_j = (1-\frac{j-1}{2\ell })r$ for $1 \le j \le \ell ,$ and note that $t_0 = r,$ $s_{\ell } = \frac{r}{2},$ and $(t_j-s_j) =\frac{r}{2\ell }.$ Therefore taking $\zeta =\frac{1}{2\ell },$ we can apply (4.15) with $s_j,t_j$ and chain the inequalities to get

4.16 $$\begin{aligned} \int _{\textrm{B}_{r/2}(x)} {\widetilde{\varphi }}\left( |\nabla ^kw_{x,r}|\right) \,\textrm{d}y \le \kappa \int _{\textrm{B}_r(x)} {\widetilde{\varphi }}\left( |\nabla ^kw_{x,r}|\right) \,\textrm{d}y + C \int _{\textrm{B}_r} {\widetilde{\varphi }}\left( \frac{|w_{x,r}|}{R^k}\right) \,\textrm{d}y, \end{aligned}$$

as required. $$\square $$

From here the proof of Theorem 4.2 proceeds analogously as in the minimising case. Note that both strong ${{\,\textrm{W}\,}}^{k,\psi }$ and ${{\,\textrm{W}\,}}^{k,\infty }$-local minimisers are *F*-extremal, so the harmonic approximation result (Lemma 3.2) can be applied to *u*. We will sketch the remaining argument, pointing out the key modifications.

### Proof of Theorem 4.2

For $\textrm{B}_r(x) \subset \textrm{B}_R(x_0)$ we will consider the slightly modified excess energy
4.17with $a_{x,r}$ as in Lemma 4.4, which satisfies $\mathrm E_M(x,r) \sim \widetilde{\mathrm E}_M(x,r)$ by convexity of $\varphi$ and (2.19). Then similarly as in Lemma 3.3, we claim that if $|\nabla ^ka_{x,2\sigma r}|,|\nabla ^ka_{x,r}|\le M$ and $\mathrm E_M(x,r)\le 1,$ then for each $\kappa , {\tilde{\delta }} \in (0,1),$ $\sigma \in \left( 0,\frac{1}{4}\right)$ we have the excess decay estimate

4.18 $$\begin{aligned} \mathrm E_M(x,\sigma r) \le C\left( \sigma + C_{\sigma }(\kappa +{\tilde{\delta }}) + C_{\sigma ,{\tilde{\delta }}}\gamma _M(\mathrm E_M(x,r)) \right) \mathrm E_M(x,r), \end{aligned}$$

where $\gamma _M$ is as in Lemma 3.2. Taking $a_1 = a_{x,r}$ we apply Lemma 3.2 to obtain the unique solution $h \in u-a_1 + {{\,\textrm{W}\,}}^{k,\varphi }_0(\textrm{B}_r(x),\mathbb R^N)$ to the problem

4.19 $$\begin{aligned} (-1)^k \nabla ^k: F''(\nabla ^k a_1)\nabla ^kh = 0 \end{aligned}$$

in $\textrm{B}_r(x).$ Then we define $a_2 = \sum _{|\alpha |\le k} \frac{\textrm{D}^{\alpha }h(x_0)}{\alpha !}(x-x_0)^{\alpha }$ as before, then we can apply Lemma 4.4 to estimate
4.20where we have used (2.20) followed by the Poincaré inequality on $\textrm{B}_r(x)$ for the first term, and the quasi-minima property (2.18) of $\nabla ^{k-1}a_{x,2\sigma r}.$ From here the rest follows by arguing exactly as in Lemma 3.3.

Given the above excess decay estimate, the theorem follows by an analogous iteration argument. A slight modification is needed to ensure that $|\nabla ^ka_{x,2\sigma r}|,|\nabla ^ka_{x,r}|\le C(M),$ which can be done using (2.19) and a similar argument as in (3.41). We also choose $\kappa ,{\tilde{\delta }}$ so that $CC_{\sigma }(\kappa +{\tilde{\delta }}) \le \frac{1}{3}\sigma ^{2\alpha }$ and the rest follows as in the proof of Theorem 1.2. $\square$

## References

[1] Acerbi, E., Fusco, N.: Semicontinuity problems in the calculus of variations. Arch. Ration. Mech. Anal. **86**(2), 125–145 (1984). 10.1007/BF00275731. (**issn: 0003-9527**)

[2] Acerbi, E., Fusco, N.: A regularity theorem for minimizers of quasiconvex integrals. Arch. Ration. Mech. Anal. **99**(3), 261–281 (1987). 10.1007/BF00284509. (**issn: 0003-9527**)

[3] Acerbi, E., Mingione, G.: Regularity results for a class of quasiconvex functionals with nonstandard growth. Ann. Della Scuola Norm. Super. Pisa - Cl. Sci. **30**(2), 311–339 (2001)

[4] Adams, R.A., Fournier, J.: Sobolev Spaces, p. 305. Academic Press, Amsterdam (2003). (**isbn: 978-0-08-054129-7**)

[5] Agmon, S., Douglis, A., Nirenberg, L.: Estimates near the boundary for solutions of elliptic partial differential equations satisfying general boundary conditions II. Commun. Pure Appl. Math. **17**(1), 35–92 (1964). 10.1002/cpa.3160170104. (**issn: 00103640**)

[6] Balci, A.K., Diening, L., Weimar, M.: Higher order Calderón-Zygmund estimates for the -Laplace equation. J. Diff Eq. **268**(2), 590–635 (2020). 10.1016/j.jde.2019.08.009. (**issn: 0022-0396**)

[7] Bärlin, M., Gmeineder, F., Irving, C., Kristensen, J.: -harmonic approximation and partial regularity, revisited (2022). arXiv:2212.12821

[8] Bhattacharya, T., Leonetti, F.: A new poincaré inequality and its application to the regularity of minimizers of integral functionals with nonstandard growth. Nonlinear Anal. Theory. Methods. App. **17**(9), 833–839 (1991). 10.1016/0362-546X(91)90157-V. (**issn: 0362546X**)

[9] Bögelein, V.: Partial regularity for minimizers of discontinuous quasi-convex integrals with degeneracy. J. Diff. Eq. **252**(2), 1052–1100 (2012). 10.1016/j.jde.2011.09.031. (**issn: 0022-0396**)

[10] Cagnetti, F.: -quasi-convexity reduces to quasi-convexity. Proc. R. Soc. Edinb. Sect. Math. **141**(4), 673–708 (2011). 10.1017/S0308210510000867. (**issn: 1473-7124, 0308-2105**)

[11] Campos Cordero, J.: Boundary regularity and sufficient conditions for strong local minimizers. J. Funct. Anal. **272**(11), 4513–4587 (2017). 10.1016/j.jfa.2017.02.027. (**issn: 00221236**)

[12] Campos Cordero, J.: Partial regularity for local minimizers of variational integrals with lower order terms. Quart. J. Math. (2021). 10.1093/qmath/haab056

[13] Carozza, M., Fusco, N., Mingione, G.: Partial regularity of minimizers of quasiconvex integrals with subquadratic growth. Ann. Mat. Pura. Ed. Appl. **175**(1), 141–164 (1998). 10.1007/BF01783679. (**issn: 0373-3114**)

[14] Carozza, M., di Napoli, A.P.: Partial regularity of local minimizers of quasiconvex integrals with sub-quadratic growth. Proc. R. Soc. Edinb. Sect. Math. **133**(6), 1249–1262 (2003). 10.1017/S0308210500002900. (**issn: 1473-7124, 0308-2105**)

[15] Chlebicka, I., Giannetti, F., Zatorska-Goldstein, A.: Elliptic problems with growth in nonreflexive Orlicz spaces and with measure or data. J. Math. Anal. App. **479**(1), 185–213 (2019). 10.1016/j.jmaa.2019.06.022. (**issn: 0022-247X**)

[16] Cianchi, A.: A Sharp Embedding Theorem for Orlicz-Sobolev Spaces. Indiana Univ. Math. J. **45**(1), 39–65 (1996). (**issn: 0022-2518**)

[17] Dal Maso, G., Fonseca, I., Leoni, G., Morini, M.: Higher-order quasiconvexity reduces to quasiconvexity. Arch. Ration. Mech. Anal. **171**(1), 55–81 (2004). 10.1007/s00205-003-0278-1. (**issn: 1432-0673**)

[18] De Filippis, C.: Quasiconvexity and partial regularity via nonlinear potentials. J. Math. Pures. Appl. **163**, 11–82 (2022). 10.1016/j.matpur.2022.05.001

[19] De Filippis, C., Stroffolini, B.: Singular multiple integrals and nonlinear potentials. J. Func. Anal. **285**(2), 109952 (2023). 10.1016/j.jfa.2023.109952

[20] De Giorgi, E.: Un esempio di estremali discontinue per un problema variazionale di tipo ellittico. Boll. Dell. Unione Mat. Ital. **4**, 135–137 (1968)

[21] Diening, L., Ettwein, F.: Fractional estimates for non-differentiable elliptic systems with general growth **20**(3), 523–556 (2008). 10.1515/FORUM.2008.027. (**issn: 1435-5337**)

[22] Diening, L., Lengeler, D., Stroffolini, B., Verde, A.: Partial Regularity for Minimizers of Quasi-convex Functionals with General Growth. SIAM Jounral Math. Anal. **44**, 3594–3616 (2012). 10.1137/120870554

[23] Diening, L., Ružička, M., Schumacher, K.: A decomposition technique for John domains. Ann. Acad. Sci. Fenn. Math. **35**, 87–114 (2010). 10.5186/aasfm.2010.3506. (**issn: 1239629X**)

[24] Diening, L., Stroffolini, B., Verde, A.: The -harmonic approximation and the regularity of -harmonic maps. J. Diff. Eq. **253**(7), 1943–1958 (2012). 10.1016/j.jde.2012.06.010. (**issn: 0022-0396**)

[25] Dong, H., Kim, D.: On -estimates for elliptic and parabolic equations with weights. Trans. Am. Math. Soc. **370**(7), 5081–5130 (2018). 10.1090/tran/7161. (**issn: 0002-9947**)

[26] Duzaar, F., Grotowski, J.F.: Optimal interior partial regularity for nonlinear elliptic systems: the method of A-harmonic approximation. manuscripta math. **103**(3), 267–298 (2000). 10.1007/s002290070007. (**issn: 1432-1785**)

[27] Duzaar, F., Mingione, G.: The p-harmonic approximation and the regularity of p-harmonic maps. Cal. Var **20**(3), 235–256 (2004). 10.1007/s00526-003-0233-x. (**issn: 1432-0835**)

[28] Duzaar, F., Steffen, K.: Optimal interior and boundary regularity for almost minimizers to elliptic variational integrals. J. Für Reine Angew. Math. Crelles J. **546**, 2002 (2002). 10.1515/crll.2002.046. (**issn: 0075-4102, 1435-5345**)

[29] Evans, L.C., Gariepy, R.F.: Blowup, compactness and partial regularity in the calculus of variations. Indiana Univ. Math. J. **36**(2), 361–371 (1987). (**issn: 0022-2518**)

[30] Evans, L.C.: Quasiconvexity and partial regularity in the calculus of variations. Arch. Ration. Mech. Anal. **95**(3), 227–252 (1986). 10.1007/BF00251360. (**issn: 0003-9527**)

[31] Franceschini, F.: Partial regularity for local minimizers. M.S. thesis, Universit’a degli Studi di Pisa (2019)

[32] Friesecke, G., James, R.D., Müller, S.: A theorem on geometric rigidity and the derivation of nonlinear plate theory from three-dimensional elasticity. Commun. Pure Appl. Math. **55**(11), 1461–1506 (2002). 10.1002/cpa.10048. (**issn: 1097-0312**)

[33] Fusco, N., Hutchinson, J.: Partial regularity of functions minimising quasiconvex integrals. Manuscripta Math. **54**(1), 121–143 (1985). 10.1007/BF01171703. (**issn: 1432-1785**)

[34] Giaquinta, M., Modica, G.: Partial regularity of minimizers of quasiconvex integrals. Ann. Inst. Henri Poincaré C. Anal. Non Linéaire **3**(3), 185–208 (1986)

[35] Gilbert, D., Trudinger, N.: Elliptic Partial Differential Equations of Second Order. Springer-Verlag, Berlin Heidelberg (1998)

[36] Giusti, E.: Direct Methods in the Calculus of Variations. World Scientific (2003). 10.1142/5002. (isbn: 978-981-238-043-2)

[37] Giusti, E., Miranda, M.: Sulla regolarità delle soluzioni deboli di una classe di sistemi ellittici quasi-lineari. Arch. Ration. Mech. Anal. **31**(3), 173–184 (1968). 10.1007/BF00282679. (**issn: 0003-9527**)

[38] Gmeineder, F.: Partial regularity for symmetric quasiconvex functionals on BD. J. de Math. Pures et App. **145**, 83–129 (2021). 10.1016/j.matpur.2020.09.005. (**issn: 0021-7824**)

[39] Gmeineder, F., Kristensen, J.: Partial regularity for BV minimizers. Arch. Ration. Mech. Anal. **232**(3), 1429–1473 (2019). 10.1007/s00205-018-01346-5. (**issn: 0003-9527**)

[40] Gmeineder, F., Kristensen, J.: Regularity for Higher Order Quasiconvex Problems with Linear Growth from Below (2019). arXiv:1903.08124

[41] Gmeineder, F., Kristensen, J.: Quasiconvex functionals of -growth and the partial regularity of relaxed minimisers. Arch. Ration. Mech. Anal. **248**, 80 (2024). 10.1007/s00205-024-02013-8

[42] Guidorzi, M.: A remark on partial regularity of minimizers of quasiconvex integrals of higher order. Univ. Degli Studi Trieste Dipartimento Sci. Mat. **33**, 1–24 (2000)

[43] Habermann, J.: Partial regularity for nonlinear elliptic systems with continuous growth exponent. Annali di Matematica **192**(3), 475–527 (2013). 10.1007/s10231-011-0233-y. (**issn: 1618-1891**)

[44] Irving, C.: -regularity results for solutions to Legendre–Hadamard elliptic systems. Calc. Var. Partial Differ. Eq. **62**, 166 (2023). 10.1007/s00526-023-02492-9

[45] Irving, C.: Regularity Theory in the Calculus of Variations and Elliptic Systems. DPhil Thesis, University of Oxford (2022)

[46] Kokilashvili, V., Krbec, M.: Weighted Inequalities in Lorentz and Orlicz Spaces. World Scientific (1991). 10.1142/1367. (isbn: 978-981-02-0612-3)

[47] Krasnosel’skii, M. A., Rutickii, Y. B.: Convex functions and orlicz spaces. P. Noordhoff (1961)

[48] Kristensen, J., Taheri, A.: Partial regularity of strong local minimizers in the multi-dimensional calculus of variations. Arch. Ration. Mech. Anal. **170**(1), 63–89 (2003). 10.1007/s00205-003-0275-4. (**issn: 0003-9527**)

[49] Kronz, M.: Partial regularity results for minimizers of quasiconvex functionals of higher order. Ann. Inst. Henri Poincaré C. Anal. Non Linéaire **19**(1), 81–112 (2002). 10.1016/S0294-1449(01)00072-5. (**issn: 02941449**)

[50] Krylov, N.V.: Parabolic and elliptic equations with VMO coefficients. Commun. Partial Differ. Equ. **32**(3), 453–475 (2007). 10.1080/03605300600781626. (**issn: 0360-5302**)

[51] Maligranda, L.: Orlicz spaces and interpolation. Seminários de Matemática (1989). (issn: 0103-5258)

[52] Maz’ya, V.G.: Examples of nonregular solutions of quasilinear elliptic equations with analytic coefficients. Funct. Anal. Appl. **2**(3), 230–234 (1969). 10.1007/BF01076124. (**issn: 0016-2663, 1573-8485**)

[53] Meyers, N.G.: Quasi-convexity and lower semi-continuity of multiple variational integrals of any order. Trans. Amer. Math. Soc. **119**, 125–149 (1965). 10.2307/1994235. (**issn: 0002-9947**)

[54] Mooney, C., Savin, O.: Some singular minimizers in low dimensions in the calculus of variations. Arch. Ration. Mech. Anal. **221**(1), 1–22 (2016). 10.1007/s00205-015-0955-x. (**issn: 0003-9527**)

[55] Morrey, C.B.: Quasi-convexity and the lower semicontinuity of multiple integrals. Pacific J. Math. **2**(1), 25–53 (1952). (**issn: 0030-8730**)

[56] Morrey, C.B.: Partial regularity results for non-linear elliptic systems. Tech. Rep. **7**, 649–670 (1968)

[57] Müller, S., Šverák, V.: Convex integration for Lipschitz mappings and counterexamples to regularity. Ann. Math. **157**(3), 715–742 (2003). 10.4007/annals.2003.157.715. (**issn: 0003-486X**)

[58] Nečas, J.: Example of an irregular solution to a nonliear elliptic system with analytic coefficients and conditions for regularity, In: R. Kluge and W. Müller, (Eds.) Theory Nonlinear Oper. Constr. Asp., pp. 197–206. Akademie-Verlag, Berlin (1977)

[59] Rao, M., Ren, Z.: Theory of Orlicz Spaces. Basel (1991)

[60] Riordan, W.: On the interpolation of operations. PhD Thesis, University of Chicago (1995)

[61] Schemm, S.: Partial regularity of minimizers of higher order integrals with -growth. ESAIM Control Optim. Calc. Var. **17**(2), 472–492 (2011). 10.1051/cocv/2010016. (**issn: 1292-8119, 1262-3377**)

[62] Schemm, S., Schmidt, T.: Partial regularity of strong local minimizers of quasiconvex integrals with -growth. Proc. R. Soc. Edinb. Sect. Math. **139**(3), 595–621 (2009). 10.1017/S0308210507001278. (**issn: 1473-7124, 0308-2105**)

[63] Simon, L.: Lectures on Geometric Measure Theory. Centre for Mathematical Analysis, Australian National University, p. 272. (1983) (isbn: 0-86784-429-9)

[64] Simon, L.: Theorems on Regularity and Singularity of Energy Minimizing Maps. Birkhäuser, Basel (1996). 10.1007/978-3-0348-9193-6 . (**isbn: 978-3-7643-5397-1 978-3-0348-9193-6**)

[65] Stampacchia, G.: The spaces and interpolation. Ann. Della. Sc. Norm Super Pisa Cl. Sci **19**(3), 443–462 (1965)

[66] Sverák, V., Yan, X.: Non-Lipschitz minimizers of smooth uniformly convex functionals. Proc. Natl. Acad. Sci. USA **99**(24), 15269–15276 (2002). 10.1073/pnas.222494699. (**issn: 0027-8424**) 12403822 10.1073/pnas.222494699 PMC137705

[67] Székelyhidi, L.: The regularity of critical points of polyconvex functionals, y Arch. Ration. Mech. Anal. **172**(1), 133–152 (2004). 10.1007/s00205-003-0300-7. (**issn: 0003-9527**)

[68] Taylor, M.E.: Partial Differential Equations I, ser. Applied Mathematical Sciences, vol. 115. Springer, New York (2011). 10.1007/978-1-4419-7055-8 . (**isbn: 978-1-4419-7054-1**)

[69] Ziemer, W.P.: Weakly Differentiable Functions, ser. Graduate Texts in Mathematics, vol. 120. Springer, New York (1989). 10.1007/978-1-4612-1015-3 . (**isbn: 978-1-4612-6985-4 978-1-4612-1015-3**)

[70] Zygmund, A.: On a theorem of Marcinkiewicz concerning interpolation of operators. J. Math. Pures Appl. **35**, 223–248 (1956). 10.1007/978-94-009-1045-4_12

